# The expression level of chicken telomerase reverse transcriptase in tumors induced by ALV-J is positively correlated with methylation and mutation of its promoter region

**DOI:** 10.1186/s13567-022-01069-2

**Published:** 2022-06-23

**Authors:** Yong Xiang, Qinxi Chen, Qingbo Li, Canxin Liang, Weisheng Cao

**Affiliations:** 1 College of Veterinary Medicine, South China Agricultural University, Guangzhou, 510642 China; 2 Key Laboratory of Zoonosis Prevention and Control of Guangdong Province, South China Agricultural University, Guangzhou, 510642 China; 3 National and Regional Joint Engineering Laboratory for Medicament of Zoonosis Prevention and Control, South China Agricultural University, Guangzhou, 510642 China; 4 Key Laboratory of Zoonosis of the Ministry of Agriculture and Rural Affairs, Guangzhou, 510642 China; 5 Key Laboratory of Veterinary Vaccine Innovation of the Ministry of Agriculture and Rural Affairs, Guangzhou, 510642 China

**Keywords:** Avian leukosis virus subgroup J, tumorigenicity, chicken telomerase reverse transcriptase, DNA methylation, promoter mutation

## Abstract

Avian leukosis virus subgroup J (ALV-J) can cause neoplastic diseases in poultry and is still widely prevalent in China. Chicken telomerase reverse transcriptase (chTERT) is the core component of telomerase, which is closely related to the occurrence and development of tumors. Our previous studies showed that chTERT is overexpressed in ALV-J tumors, but the mechanism is still not completely clear. Therefore, this study aims to analyze the possible molecular mechanism of chTERT overexpression in ALV-J tumors from the perspective of DNA methylation and promoter mutation. Methylation sequencing of the chTERT amplicon showed that ALV-J replication promoted the methylation level of the chTERT promoter. And the methylation level of the chTERT promoter in ALV-J tumors was significantly higher than that in tumor-adjacent and normal tissues. Compared with the tumor-adjacent and normal tissues, the chTERT promoter in each ALV-J tumors tested had a mutation of −183 bp C > T, and 36.0% (9/25) of the tumors also had mutations of −184 bp T > C, −73 bp::GGCCC and −56 bp A > T in the chTERT promoter, which formed the binding sites for the transcription factors NFAT5, TFAP2A and ZEB1, respectively. The results of RT–qPCR and Western blotting showed that the occurrence of these mutations significantly increased the expression level of chTERT. In conclusion, this study demonstrated that the high expression of chTERT in ALV-J tumors is positively correlated with the level of hypermethylation and mutation in its promoter, which provides a new perspective for further research on the molecular mechanism of chTERT in ALV-J tumorigenesis.

**Supplementary Information:**

The online version contains supplementary material available at 10.1186/s13567-022-01069-2.

## Introduction

Avian leukosis (AL) is caused by avian leukosis virus (ALV), which can induce a variety of neoplastic diseases in poultry. The disease has various clinical manifestations, including myeloma, lymphoma, hemangioma, and fibrosarcoma [1]. It is a kind of provenance disease that seriously harms the poultry industry worldwide [2]. Since the 1990s, AL has caused significant economic losses to China’s chicken industry, especially avian leukosis virus subgroup J (ALV-J) [3]. It seriously harms the healthy development of white-feathered broilers, egg-type broilers, yellow-feathered broilers and local broilers and is still widely prevalent in China [4].

Telomerase reverse transcriptase (TERT), as the core component of telomerase regulation, is closely related to the development of cancer and cell growth [5]. Significantly elevated expression levels of human TERT (hTERT) have been found in most human malignancies, such as sarcomas [6], brain tumors [7], colorectal tumors [8], and breast cancer [9], increasing the risk of tumor recurrence. Studies have shown that mutations and methylation in the hTERT promoter region play a crucial role in the regulation of hTERT transcription levels and telomerase activity [10–13]. Preliminary studies by our group have shown that the chicken TERT (chTERT) gene is one of the main integration sites for ALV-J provirus to insert into the host genome, and it is significantly overexpressed in ALV-J tumors [14]. However, the reasons for its high expression are still not completely clear.

5'-Methylcytosine (5mC) is the main form of DNA methylation and one of the earliest and most thoroughly studied epigenetic regulatory mechanisms, playing an important role in cancer, gene expression, aging, atherosclerosis, Alzheimer’s disease and other diseases [15–17]. Studies have shown that the increased expression of hTERT in colorectal cancer and gastric cancer was associated with the degree of hypermethylation of the hTERT gene, which seriously affected recurrence after treatment [18, 19]. In patients with liver cancer, the methylation level of the hTERT promoter in cancer tissues was significantly higher than that in tumor-adjacent tissues, and the expression level of the hTERT gene was also increased by tens of times [20]. However, it has been reported that when CpG islands with low methylation overlie the promoter region of the gene, telomerase activity is inhibited, which is inconsistent with a previous study showing that methylation in the promoter region of the hTERT gene promoted its gene expression level [11, 21]. In conclusion, the mechanisms by which methylation in the TERT promoter region regulates gene expression in different tumor diseases are not entirely the same and may be methylation-dependent or methylation-independent, requiring precise and targeted studies. The 5' region of the chTERT gene and its promoter region are part of a large CpG island of approximately 4 kb, suggesting that chTERT expression may be regulated by methylation [22]. Therefore, we were inspired to actively explore whether there are significant differences in the methylation levels of the chTERT gene and its promoter region in tumors induced by ALV-J compared with those in tumor-adjacent tissues or normal tissues, as well as the methylation effects on chTERT gene expression and telomerase activity.

The formation of many tumors is inseparable from the occurrence of genetic mutations, but previous studies have shown that genetic mutations mostly occur in coding regions. However, a study found that there was a high frequency of mutations in the hTERT promoter region in melanoma, reaching approximately 70.0%, and the mutation sites were concentrated in −57 bp T > G, −124 bp G > A (C228T) and −146 bp G > A (C250T). After the mutation, the sequence of GGAA/T or CCGGAA/T was obtained, forming a new transcriptional binding site for the Ets ternary complex factor, thereby actively participating in the regulation of hTERT expression and promoting the occurrence and development of tumors [23, 24]. Subsequently, two popular mutation sites, C228T and C250T, were also found in adrenal tumors, which can promote the expression of hTERT and increase the activity of telomerase, providing a suitable environment for tumor growth [25]. In addition, scientists have successively found mutations in the hTERT promoter region in thyroid cancer [26], liposarcoma [27], hepatocellular carcinoma [28], urothelial cancer and other tumors [29, 30], but the mutation sites and mutation frequencies were not the same. Since the first discovery of TERT promoter mutations in melanoma, an increasing number of studies have shown that TERT promoter mutations play an important role in tumorigenesis [31, 32]. However, most of the current studies on TERT promoter mutations focus on human tumor diseases, and there are few studies on chicken-related tumor diseases. Therefore, it is of great significance to identify the mutation of the chTERT promoter region and its regulatory effect on gene expression in ALV-J-induced tumors in a timely manner.

In this study, DF-1 cells and LMH cells were used as in vitro models. The methylation and mutation characteristics of the chTERT promoter region in chicken tumor cells and normal cells were determined by methylation sequencing and Sanger sequencing, and the effect of ALV-J replication on the chTERT promoter methylation level was analyzed. ALV-J tumor tissues and tumor-adjacent and normal tissues were used as research objects to investigate the methylation and mutation characteristics of the chTERT promoter region in ALV-J tumors. Moreover, the influence of these differences on chTERT expression was analyzed statistically. It is expected that this study can preliminarily answer the scientific question of how the high expression of chTERT was formed in ALV-J tumors and provide a useful reference and theoretical support for the research of other avian tumor diseases.

## Materials and methods

### Cells, virus, ALV-J tumors and antibodies

The avian leghorn male hepatoma cell line LMH and chicken fibroblast cell line DF-1 were maintained in our laboratory and cultured in DMEM/F12 or DMEM (Gibco, Thermo Fisher Scientific, Inc., Grand Island, NY, USA) supplemented with 10% fetal bovine serum (FBS) and maintained at 37 °C with 5% CO_2_. The ALV-J Hc1 strain was maintained in our laboratory. Twenty-five tumor tissue samples induced by chronic transformed ALV-J and their corresponding tumor-adjacent and normal tissues were confirmed and collected from our previous ALV-J artificial tumorigenicity experiment and clinical tumor cases in large-scale breeding poultry farms (Additional file 1). The chicken single factor serum of chTERT was prepared and preserved by our laboratory in the early stage. The anti-GAPDH primary monoclonal antibody was purchased from Abcam, Inc. (Cambridge, UK). IRDye^®^ 800CW goat anti-rabbit IgG and donkey anti-chicken secondary antibodies were purchased from LI-COR Biosciences, Ltd. (Nebraska, USA).

### Prediction of chTERT promoter methylation

**Figure 1:**
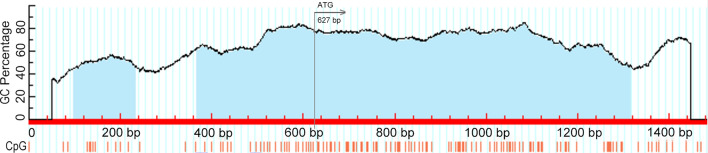
**Prediction of DNA methylation in the chTERT promoter region.**

We referred to the related sequences of the chTERT promoter region and coding region on NCBI (GenBank: EU650197.1, NM.001031007) by using the online software MethPrimer to analyze the possible methylation CpG sites in the chTERT promoter region. The results (Figure 1) showed that there were 2 CpG islands from 600 bp upstream to 800 bp downstream of ATG of the initiation codon, which contained more than 110 CpG sites. Therefore, in this study, this sequence was used as a target gene for DNA methylation analysis in the chTERT promoter region, and the position of the amplicon relative to the ATG initiation codon in the chTERT coding region was −627 bp to + 873 bp.

### Target gene bisulfite sequencing

DNA was extracted from cells or tissue samples according to the instructions of the DNA extraction kit (Omega, Norcross, GA, USA). Target gene bisulfite sequencing was conducted by E-Gene Co., Ltd. (Shenzhen, China). Briefly, primers (Table 1) were designed to recognize regions without CpG sites to avoid amplification bias of methylated versus unmethylated sequences. Bisulfite sequencing PCR (BSP) validation experiments were conducted as follows: 500 ng of genomic DNA was converted using a ZYMO EZ DNA Methylation-Gold Kit™ (Zymo Research, California, USA) according to the manufacturer’s instructions. After purification of the converted products, PCR amplification was carried out in a final reaction volume of 50 µL consisting of 3 µL purified conversion fractions, 4 µL 2.5 mM dNTP, 5 µL 10× buffer, 1 µL BSP primers, 0.5 µL JumpStart™ Taq DNA Polymerase (Sigma-Aldrich, St. Louis, Missouri, USA) and 36.5 µL water and the following thermal cycling program was 94 °C 1 min, 30 cycles of 94 °C 10 s, 58 °C 30 s, 72 °C 30 s then extension of 5 min at 72 °C and products were held at 12 °C. After amplification, the PCR products were further used for library construction, and the final libraries were quantified by an Agilent 2100 Bioanalyzer (Agilent Technologies, California, USA) and real-time PCR assay and then sequenced by Illumina HiSeq.

**Table 1:** **BSP Primer sequences for methylation analysis of the chTERT amplicon**

Primers position (bp)^1^	Sequences (5′-3′)	Annealing temperature	Product (bp)
−579-F^2^	TTTTTTTTATTAAATTGTGTTATTG	65 ℃	194
−386-R^3^	CTCTTACTTTATCCCTAAAAAAACC		
−315-F	AGTTTAATTGTTAATTTATTTTTATTT	64 ℃	159
−157-R	TTAAATTTAAAAACAATTTCTTCTC		
−167-F	TTAAATTTAATTTGAGTTTTTTTTAG	69 ℃	323
+ 156-R	ATACCACCCTCCTACAACC		
+ 263-F	TTTGTTTTTAGTAGGTAGGGAGGAG	67 ℃	267
+ 529-R	AACCCCAAACATACAAATCTTTAAC		
+ 576-F	TTGGGAGGGAAGTATTATTTTTTTT	67 ℃	264
+ 869-R	CCCTTTTAATCCTACTCCTCATACC		

^1^The primer position is relative to the start codon ATG, and upstream of the start codon is marked as “−”, while ATG and its downstream regions are marked as “ + ”

^2^“F” stands for upstream primer

^3^“R” stands for downstream primer

### PCR amplification and Sanger sequencing of the chTERT promoter region

PCR was used to amplify an approximately 1000 bp fragment at the end of the chTERT promoter region (approximately 1000 bp upstream of ATG) to analyze its mutation characteristics. The primer sequences (5’-GTTGGTGGTATGGCAGTA-3′, 5′-TCCTCCCGCGCTACATTG-3′) were used for amplification, and the annealing temperature was 57 °C. Phanta^®^ Max DNA Polymerase (Vazyme, Nanjing, China) was used for amplification. The products were detected by 1% agarose gel electrophoresis in 1 × TAE with EB staining, and the PCR product was recovered by a Gel Extraction Kit (Omega) and cloned into the pMD-18 T vector according to the manufacturer’s instructions (Takara, Tokyo, Japan). After that, recombinants were transformed into E. coli DH5α competent cells (Takara), and monoclonal clones were selected on LB plates containing ampicillin. DNA was extracted from positive clones using a Plasmid Mini Kit (Omega) and sent to Sangon Biotech (Shanghai, China) for Sanger sequencing.

### Real-time fluorescence quantitative PCR (RT–qPCR)

The tissue samples were homogenized by a cryogenic grinder, and then the total RNA was extracted from tissues with TRIzol reagent (Fastagen Biotech, Shanghai, China) according to the manufacturer’s recommendations. cDNA was synthesized from the total RNA template with random primers using a PrimeScript RT Reagent kit (TaKaRa). The RT–qPCR was performed using Hieff^®^ qPCR SYBR Green Master Mix (YEASEN, Shanghai, China) on a CFX96TM Real-time PCR System (Bio–Rad, California, USA). Expression levels were quantified using the 2^−ΔΔCt^ method and normalized to GAPDH expression. The sequences of the primers used were the same as those used in our previous study [33].

### Western blotting

The tissue samples were homogenized at low temperature, after which NP40 lysis buffer (Beyotime, Shanghai, China) was used to extract protein. The concentration of extracted protein was determined by the BCA Protein Assay Kit (Beyotime). Subsequently, equal amounts of total protein were separated by SDS–polyacrylamide gel electrophoresis (SDS–PAGE) (Beyotime) and then transferred onto a nitrocellulose membrane. The membranes were immunoblotted with primary antibodies at 4 °C overnight followed by a corresponding secondary antibody at 37 °C for 1 h. Finally, the blots were scanned using an Odyssey Infrared Imaging System (LI-COR, Nebraska, USA).

### Statistical analysis

All the results are presented as the means ± standard deviations. Statistical analysis was performed by Student’s *t* test using GraphPad Prism software, and a *P* value of < 0.05 was considered significant.

## Results

### The methylation level of the chTERT promoter region in LMH cells is higher than that in DF-1 cells

**Figure 2:**
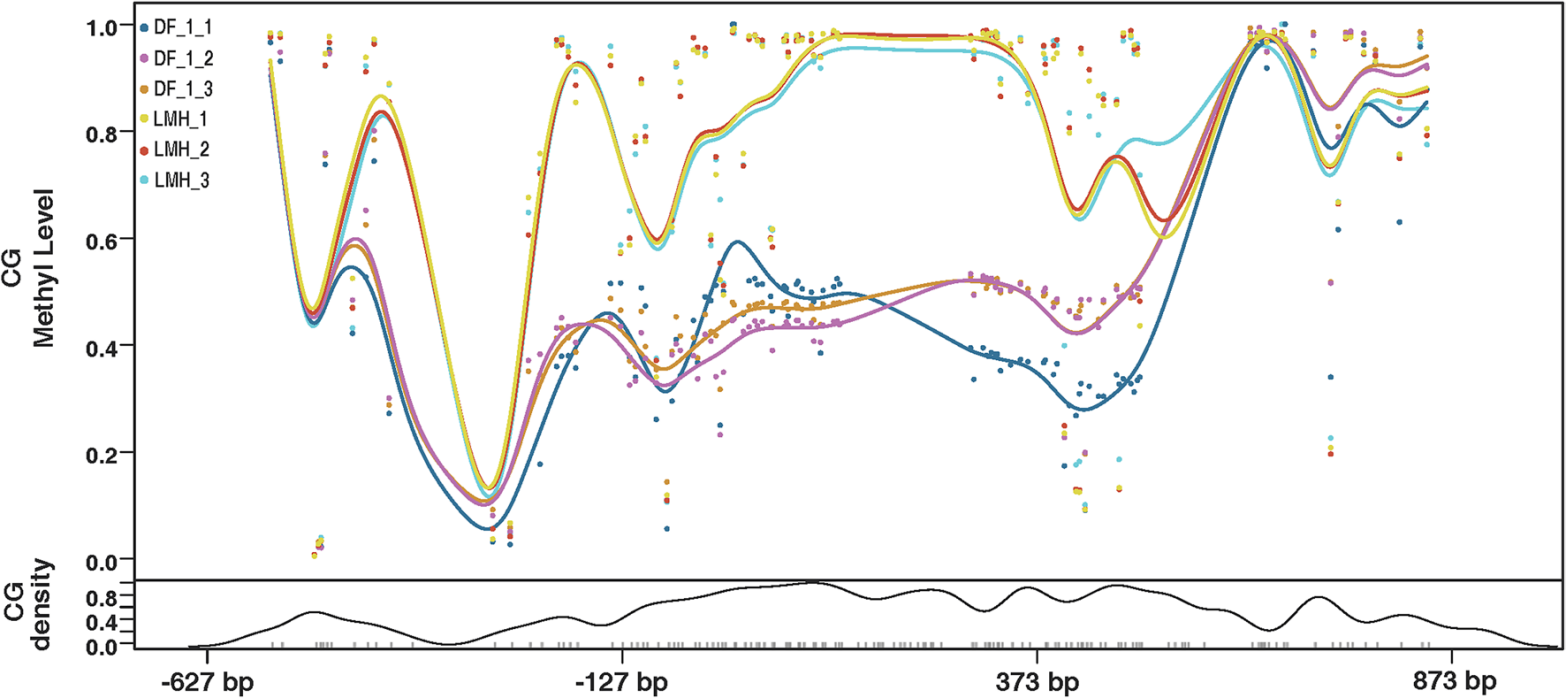
**Comparison of chTERT promoter methylation levels in LMH and DF-1 cells.**

LMH and DF-1 cells were used as in vitro models of chicken tumor cells and healthy cells, respectively, to analyze the difference between them in methylation levels of the chTERT promoter region. The results showed that the methylation level of the chTERT promoter region in LMH cells was higher than that in DF-1 cells (Figure 2). Our previous study showed that telomerase activity was positive in LMH cells and negative in DF-1 cells [33]; that is, chTERT was not expressed in DF-1 cells. In conclusion, the expression of chTERT in LMH cells is related to the hypermethylation level of the promoter region.

### Replication of ALV-J promotes methylation of the chTERT promoter region

**Figure 3:**
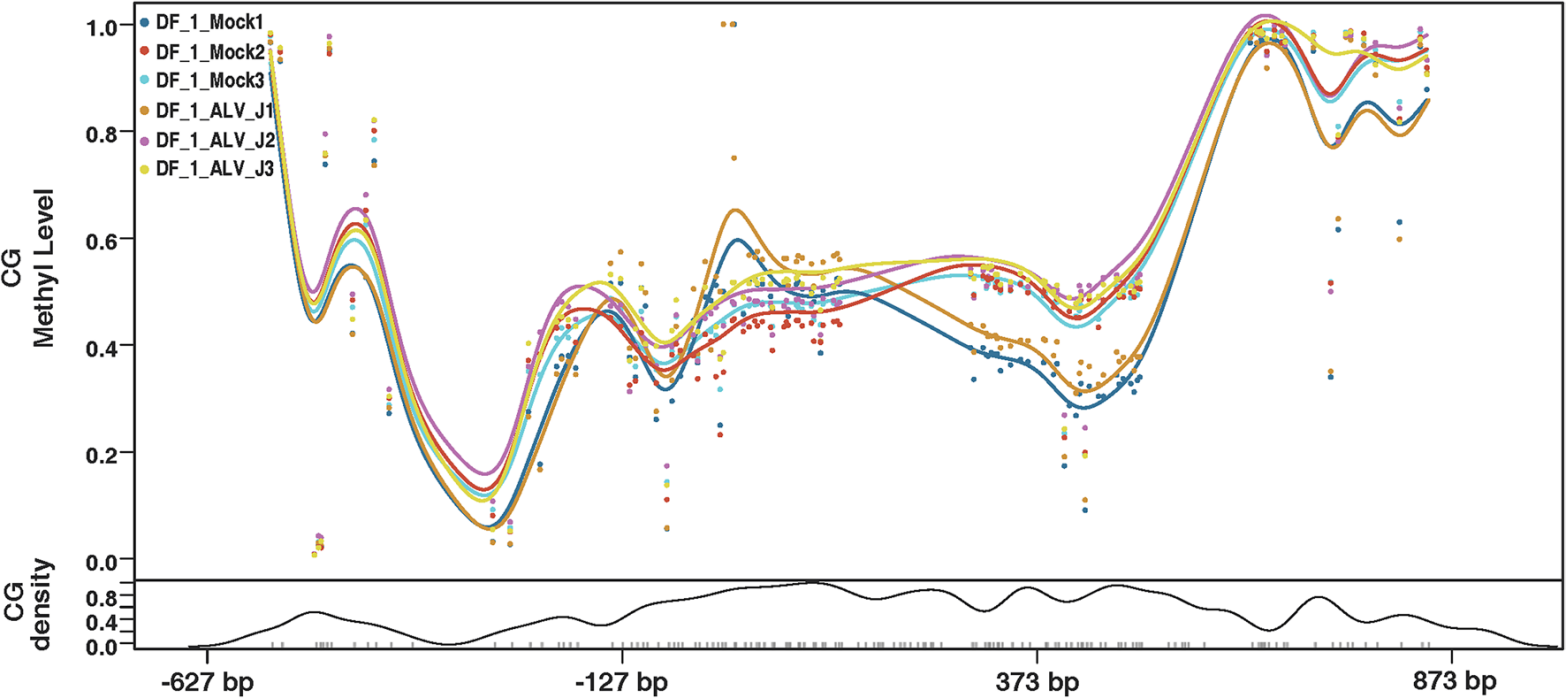
**Replication of ALV-J promotes methylation of the chTERT promoter region in DF-1 cells.**

**Figure 4:**
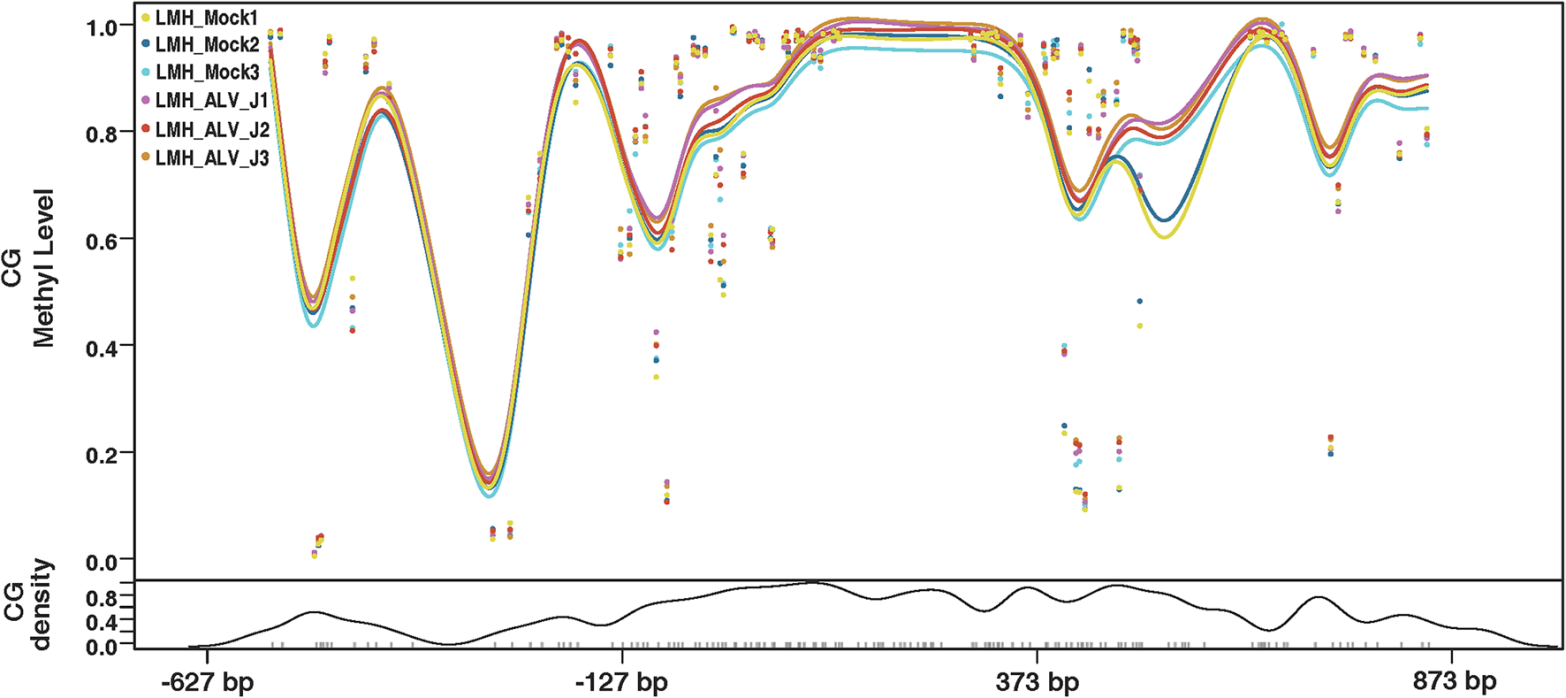
**Replication of ALV-J promotes methylation of the chTERT promoter region in LMH cells.**

**Figure 5:**
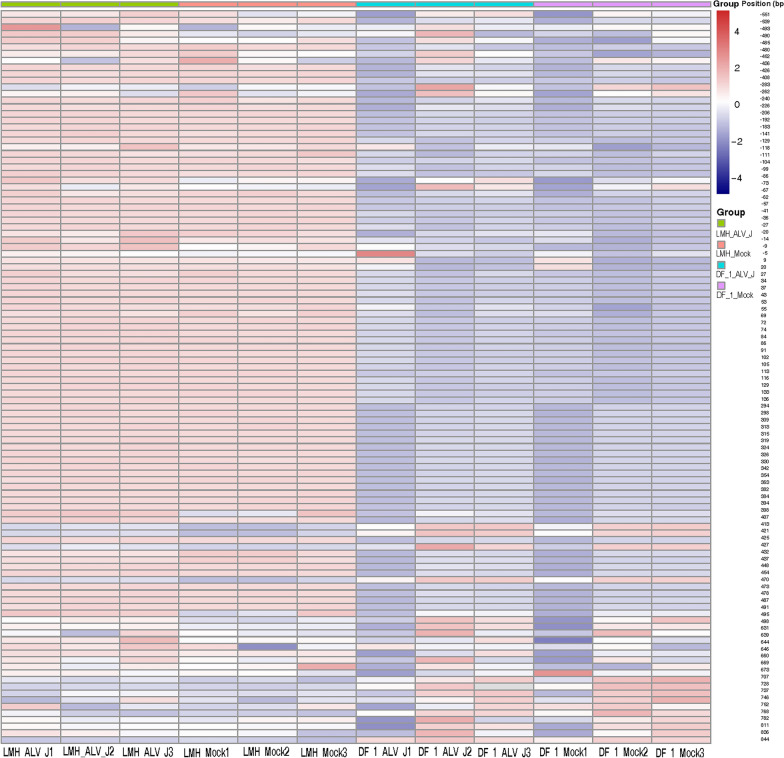
**Heatmap of the DNA methylation level of the chTERT amplicon in LMH cells and DF-1 cells.**

LMH cells and DF-1 cells were infected with ALV-J at 1.0 multiplicity of infection (MOI) and maintained for 7 days, and the cell DNA was extracted for chTERT amplicon methylation sequencing. The results showed that ALV-J replication promoted the methylation of the chTERT promoter region in both DF-1 cells (Figure 3) and LMH cells (Figure 4), and the thermal map of the methylation level is shown in Figure. 5, the corresponding methylation matrix data are shown in Additional file 2. Our previous study showed that the replication of ALV-J upregulated the expression level of chTERT [33]. In conclusion, the expression level of chTERT is positively correlated with the methylation level of its promoter region.

### The high expression of chTERT in ALV-J tumors correlates with the high methylation level of its promoter region

**Figure 6:**
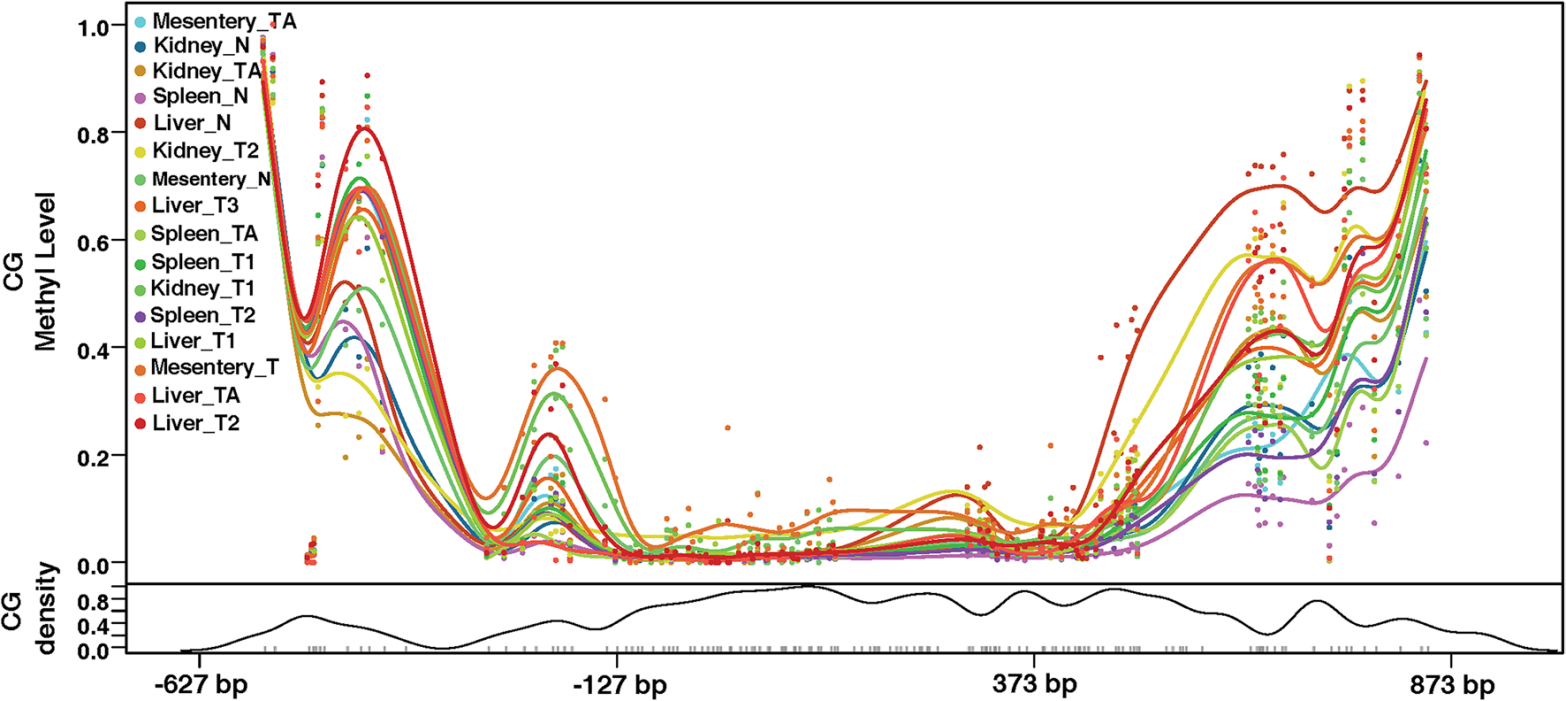
**The DNA methylation level change curve of the chTERT amplicon in ALV-J tumors and tumor-adjacent and normal tissues.** T: tumor tissues; TA: tumor-adjacent tissues; N: normal tissues.

**Figure 7:**
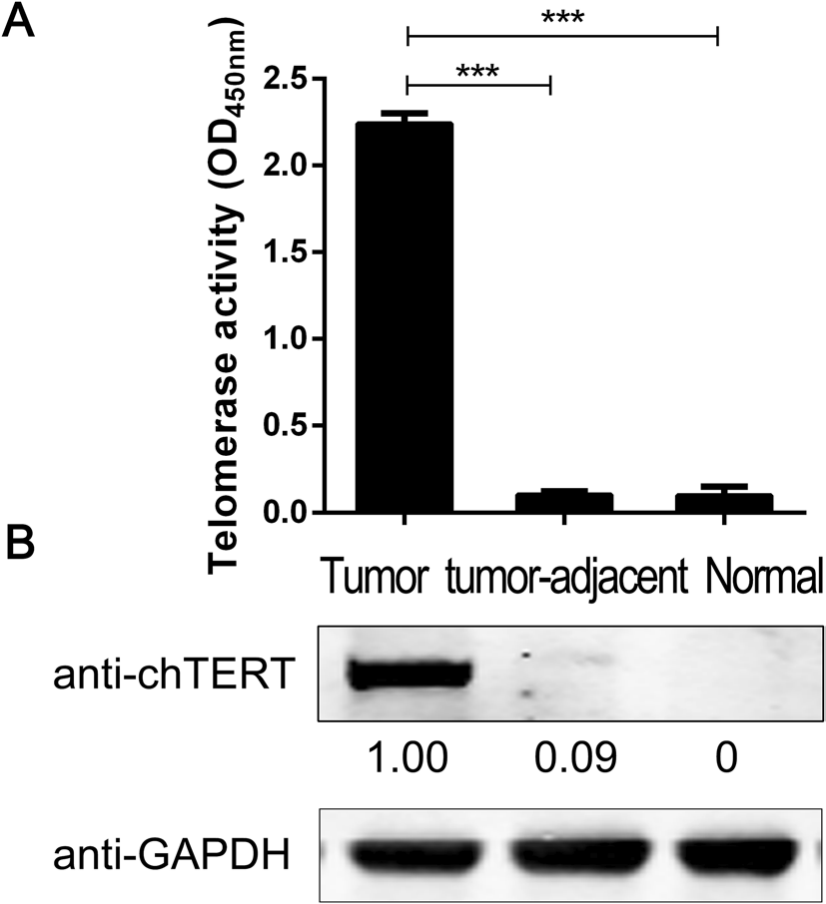
**chTERT was significantly highly expressed in ALV-J tumor tissues.** Analysis of telomerase activity (**A**) and chTERT protein expression levels (**B**) in tumor tissue, tumor-adjacent and normal tissues.

**Figure 8:**
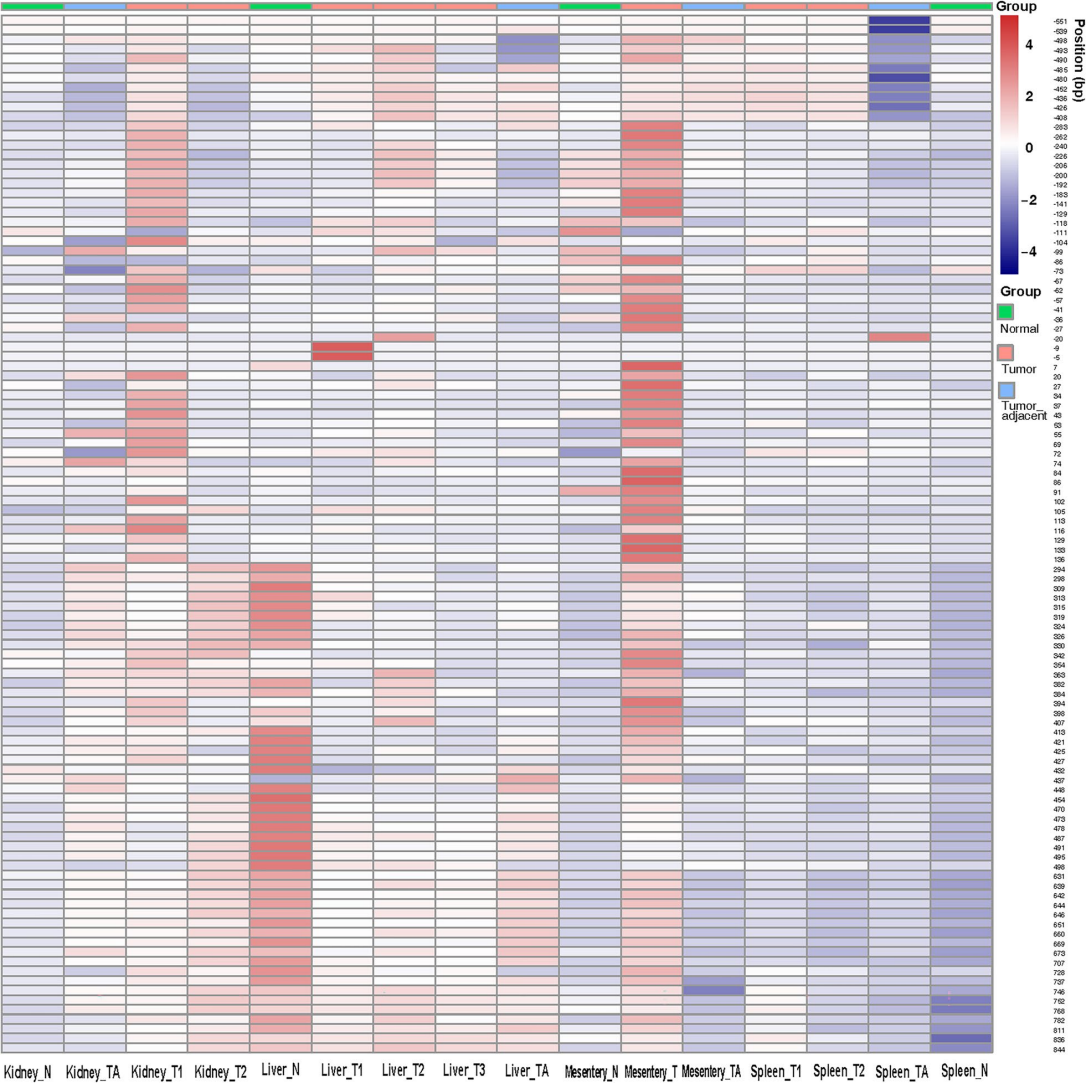
**Heatmap of the DNA methylation level of the chTERT amplicon in ALV-J tumors and tumor-adjacent and normal tissues.** T: tumor tissues; TA: tumor-adjacent tissues; N: normal tissues.

Some of the ALV-J tumor tissues were selected for methylation sequencing of the chTERT promoter region. The results showed that the methylation level of the chTERT promoter region in ALV-J tumor tissue was higher than that in tumor-adjacent tissues and normal tissues (Figure 6), suggesting that the high expression of chTERT in ALV-J tumor tissues (Figure 7) was related to the high methylation level of its promoter region; the heatmap of its methylation level is shown in Figure 8, the corresponding methylation matrix data are shown in Additional file 3.

### Mutation of the chTERT promoter region promotes its methylation and forms binding sites for the transcription factors ZEB1, TFAP2A and NFAT5

The sequence of approximately 1000 bp at the end of the chTERT promoter region was amplified by PCR (Additional file 4), and Sanger sequencing was performed. Then, the sequences were compared to find a unified rule. The results showed (Additional file 5) that there were no uniform mutations in the chTERT promoter region in tumor-adjacent tissues compared with normal tissues. However, compared with tumor-adjacent or normal tissues, the −183 bp of the chTERT promoter region in all ALV-J tumor tissues was mutated from T to C (−183 bp C > T). In addition, nine tumor tissues (*n* = 25) were further mutated; that is, the chTERT promoter at the −184 bp site was mutated from C to T (−184 bp T > C), and the −56 bp was mutated from T to A (−56 bp A > T). Moreover, after −73 bp in the chTERT promoter region of this part of the tumor tissue, there was the insertion of 5 bases GGCCC (−73 bp::GGCCC). In this study, tumor tissues with further mutations in this region were called Mutant, and those without such mutations were called Wild type.

**Figure 9:**
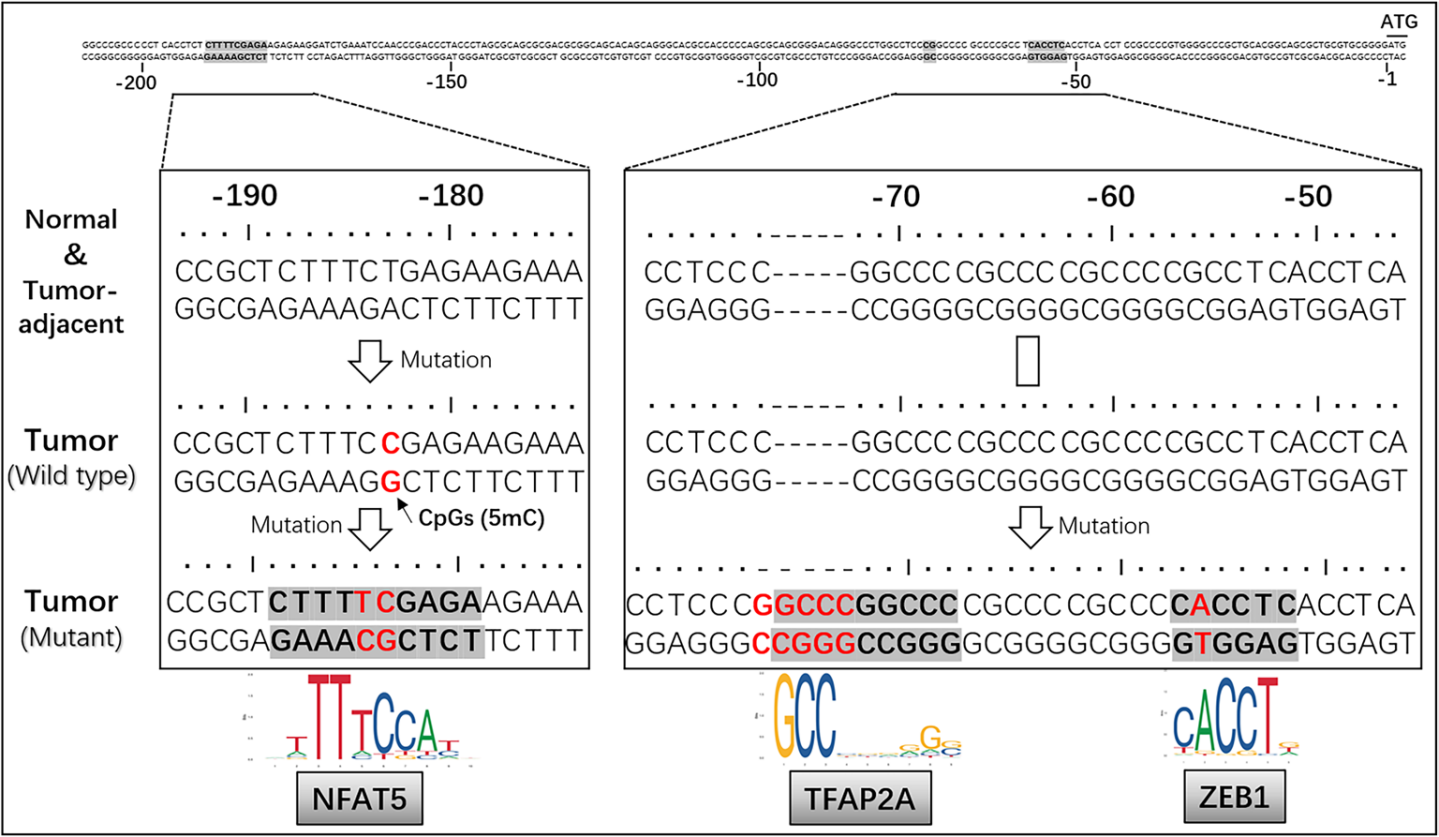
**Schematic diagram of mutations in the chTERT promoter region in ALV-J tumors relative to its tumor-adjacent and normal tissues.**

Further analysis showed that the mutation of −183 bp C > T in ALV-J tumor tissue makes this site a CpG site that may be methylated. As seen from Figure 9, the methylation level of −183 bp in the chTERT promoter region of some tumor tissue samples (such as Kidney_T1, Liver_T2, Liver_T3, and Mesentery_T) is indeed higher than that of the tumor-adjacent and normal tissues. This finding indirectly suggests that mutations at this site may promote chTERT expression through methylation. On this basis, the sequences before and after the mutation were imported into the JASPAR database for analysis, and it was found that the mutation or insertion of −184 bp T > C, −73 bp::GGCCC and −56 bp A > T forms the binding sites of transcription factors of nuclear factor of activated T cells 5 (NFAT5), transcription factor AP-2a (TFAP2A) and E box zinc finger E-box binding homeobox 1, ZEB1), respectively.

### The high expression of chTERT in ALV-J tumors correlates with its promoter mutation

**Figure 10:**
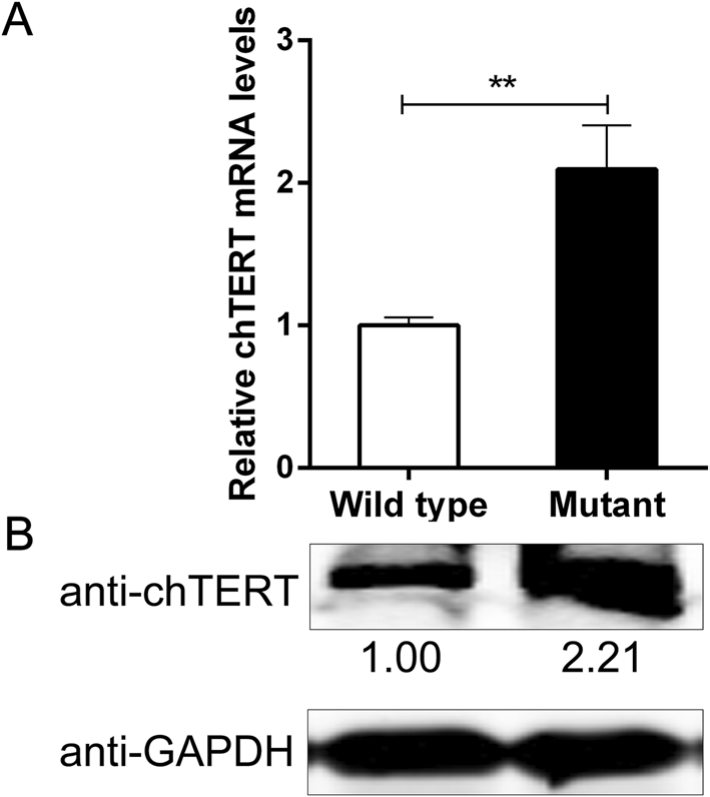
**The expression levels of chTERT mRNA (A) and protein (B) in tumor tissues with the wild-type or mutant chTERT promoter.**

To understand how the mutation of the chTERT promoter region in ALV-J tumor tissues affects the expression of chTERT, the expression of chTERT in wild-type and mutant tumors was analyzed by Western blot and RT–qPCR. The results showed that the mRNA and protein expression levels of chTERT in mutant tumor tissues were significantly higher than those in wild-type tissues (Figure 10, *p* < 0.01), suggesting that the high expression of chTERT in ALV-J tumors was also significantly related to the mutation of its promoter region.

## Discussion

Studies have shown that transcriptional reactivation of TERT is associated with methylation and mutation of its promoter region in a variety of human tumor diseases [34–37]. However, there is no relevant research demonstrating the difference in the methylation level of chTERT in the promoter region of ALV-J-induced tumors, nor whether there are mutations in the promoter region or the characteristics of the mutations. Therefore, this study focused on the chTERT promoter region to analyze the methylation and expression of this promoter region in the LMH tumor cell line and DF-1 normal cell line, as well as in ALV-J tumor tissues, tumor-adjacent tissues and normal tissues, and to explore the effects of these differences and characteristics on chTERT expression.

In this study, the differences in chTERT promoter methylation levels between LMH cells and DF-1 cells were analyzed in vitro, and the results showed that the methylation levels of the chTERT promoter in telomerase-positive LMH cells were significantly higher than those in telomerase-negative DF-1 cells, but the exact reason for this is still unknown. After reviewing the literature, we speculated that this might be related to the different immortalization mechanism of the two cells. DF-1 cells are telomerase-negative immortalized cell lines, which are not cancer cells in the strict sense, and their immortal proliferation does not depend on the expression of chTERT, but on another mechanism, namely the alternative lengthening of telomeres [38]. LMH cells are telomerase-positive chicken tumor cell lines, and their immortal proliferation depends on the expression of telomerase or chTERT. In order to obtain continuous and stable expression of telomerase activity, the chTERT promoter has a high level of methylation. These results suggested that the reactivation of telomerase in LMH cells was related to the high methylation level of the chTERT promoter region.

To understand the effect of ALV-J replication on chTERT promoter methylation, ALV-J was inoculated with 1.0 MOI on DF-1 and LMH cells. The results showed that after ALV-J inoculation, the methylation level of the chTERT promoter region consistently showed an increasing trend. Our previous research showed that replication of ALV-J upregulated the expression of chTERT and telomerase activity, indicating that methylation in the chTERT promoter region could promote its expression. This was further confirmed by comparing the methylation levels of the chTERT promoter region in ALV-J tumor tissues, tumor-adjacent tissues and normal tissues. That is, the methylation level of the chTERT promoter region in tumor tissues was higher than that in tumor-adjacent and normal tissues, which was positively correlated with the expression of chTERT. This is consistent with the conclusions of studies on human small-cell lung cancer, thyroid cancer, hepatocellular carcinoma and other cancer diseases [20, 39, 40]. Imperfectly, allele specific methylation of chTERT promoter was not analyzed in this study. Indeed, this is because this research was not designed in a more comprehensive way. Secondly, due to the large number of chickens, which are economic animals, the sources of tumor samples are complicated, it is less feasible to trace their genetic information and lack of database support. Therefore, if based on this research to supplement the experimental analysis of the allele specific methylation, its feasibility is worthy to be considered, but it can be further explored and analyzed in detail in our subsequent new research. Allele specific methylation analysis is interesting, but we believe that the lack of this data may not affect the main thrust of this study.

Previous studies have shown that when the insertion of chronically transformed ALV-J is integrated near the proto-oncogene of the host gene, its insertion integration site is usually located ± 2500 bp away from the transcription start site [14]. Therefore, to avoid the influence of ALV-J insertion and integration as much as possible and to analyze the mutation of the methylated region in the chTERT promoter region and for the convenience of the experiment, PCR amplification was only performed on the gene sequence 1000 bp upstream of ATG at the end of the promoter region to analyze the mutation characteristics. The results showed that there was no difference in the promoter sequence between tumor-adjacent tissues and normal tissues. Compared with tumor-adjacent and normal tissues, ALV-J tumors have more significant mutations in the promoter sequence of this segment, and all have the mutation of −183 bp C > T. On this basis, some tumor tissues also have the mutation of −183 bp C > T, 184 bp T > C, −73 bp::GGCCC and −56 bp A > T; these mutation sites are not identical to the mutation hotspots of C228T and C250T in the hTERT promoter region in human tumor diseases [25, 41, 42] because in different tumor diseases, the mutation frequency and mutation sites of TERT are not completely consistent. On the other hand, this study also attempted to compare the mutation of the chTERT promoter region in LMH cells and DF-1 cells, but no uniform mutation pattern was found after comparison, which was not consistent with the mutation characteristics of chTERT in ALV-J tumors or tumor-adjacent and normal tissues. However, this was not difficult to understand, because even the corresponding cells in vitro cannot completely simulate the pathological model at the tissue level in vivo, there must be differences.

Combined with the results of methylation sequencing, it was found that the mutation of −183 bp C > T makes this site a CpG site that may be methylated. It has also been proven that methylation at this site in the chTERT promoter region has indeed occurred in some tumor tissues. On this basis, the JASPAR database was used to analyze the differences caused by the sequence differences before and after the mutation. The results showed that the mutations of −184 bp T > C, −73 bp::GGCCC and −56 bp A > T formed transcriptional binding sites that could be bound by the transcription factors NFAT5, TFAP2A and ZEB1, respectively. Western blot and RT–qPCR analyses showed that the expression of chTERT in mutant tumor tissues was significantly higher than that in wild-type tumor tissues. This is because the mutation of −183 bp C > T promotes an increase in the methylation level, thereby upregulating the expression of chTERT. On the other hand, it is presumed to be regulated by the transcription factors NFAT5, TFAP2A and ZEB1.

After reviewing the literature, it was determined that NFAT5 has similar functions to TERT and plays an important role in various activities, such as immune regulation, metabolic regulation, DNA damage repair and tumorigenesis [43–45], such as non-small-cell lung cancer [46], pancreatic cancer [47], chronic lymphocytic leukemia [48] and melanoma [49]. It plays an important role in the occurrence and development of various human tumor diseases, and studies have shown that NFAT5 can regulate the transcription of the mouse TERT gene and promote the expression of TERT [50]. TFAP2A is closely related to the process of regulating the cell cycle, epithelial-mesenchymal transition and apoptosis and can participate in regulating the proliferation and migration of cervical cancer [51], ovarian cancer [52] and other tumor cells and promote the occurrence and development of tumors. As a transcriptional regulator containing multiple functional domains, ZEB1 can participate in the regulation of the expression of various oncogenes, including hTERT; for example, ZEB1 can promote the occurrence and development of breast cancer by upregulating the expression of hTERT [53]. In colorectal cancer, the hTERT/ZEB1 complex directly regulates E-cadherin to promote EMT [54]. This study speculates that one of the main reasons why the expression of chTERT in mutant tumor tissues is significantly higher than that in wild-type tumor tissues is that NFAT5, TFAP2A and ZEB1 are actively involved in regulating the expression of chTERT, but their interaction with chTERT and the regulatory mechanism remain to be further elucidated, which also provides new scientific questions and research directions for future generations to further study the mechanism of chTERT in ALV-J-induced tumorigenesis.

In summary, this study demonstrated that the high expression of chTERT in ALV-J tumors was positively correlated with the hypermethylation and mutation level of its promoter region, and we analyzed the molecular mechanism by which chTERT promotes the tumorigenicity of ALV-J from the perspective of DNA methylation and promoter mutation. This study provides a new perspective for further research on the role and molecular mechanism of chTERT in tumorigenicity by ALV.

## Supplementary Information

**Additional file 1.** **ALV-J Tumor tissues and their sources and quantities.**

**Additional file 2.** **Matrix of methylation levels of chTERT amplicon CG sites in LMH cells and DF-1 cells.**

**Additional file 3. Matrix of methylation levels of chTERT amplicon CG sites in ALV-J tumor, tumor-adjacent and normal tissues.**

**Additional file 4. Agarose gel electrophoresis of PCR amplification of the chTERT promoter region.** Lanes 1-8: different tissue samples; Lane 9: negative control.

**Additional file 5. Analysis of DNA sequence mutations in the promoter region of chTERT.** Green box: normal tissues; blue box: tumor-adjacent tissues; red box: tumor tissues; orange box: mutant tumors. T: tumor tissues; TA: tumor-adjacent tissues; N: normal tissues.

## Abbreviations

chTERT: chicken telomerase reverse transcriptase
hTERT: human telomerase reverse transcriptase
AL: avian leucosis
ALV: avian leukosis viruses
ALV-J: avian leukosis virus subgroup J
TRAP: telomeric repeat amplification protocol
MOI: multiplicity of infection
RT–qPCR: real-time quantitative PCR
5mC: 5'-Methylcytosine
FBS: fetal bovine serum
BSP: bisulfite sequencing PCR
NFAT5: nuclear factor of activated T cells 5
TFAP2A: transcription factor AP-2a
ZEB1: E box zinc finger E-box binding homeobox 1

## Declarations

### Availability of data and materials

The data on which the conclusions of the manuscript rely are presented in the main paper and supplementary information.
