# Supplementary material for: The expression level of chicken telomerase reverse transcriptase in tumors induced by ALV-J is positively correlated with methylation and mutation of its promoter region
Source: Vet Res. 2022 Jun 23;53:49. doi: 10.1186/s13567-022-01069-2 (PMC9229480; doi:10.1186/s13567-022-01069-2)
Supplement: Supplementary file 1 — Additional file 1. ALV-J Tumor tissues and their sources and quantities. [file 13567_2022_1069_MOESM1_ESM.doc]

**Additional file 1 ALV-J Tumor tissues and their sources and quantities**

| Tumor tissues | Source and quantity of tissue samples | | Total |
| --- | --- | --- | --- |
|  | Artificial tumorigenic | Clinical cases |  |
| Heart | 1 | 0 | 1 |
| Liver | 1 | 12 | 13 |
| Spleen | 0 | 4 | 4 |
| Kidney | 1 | 2 | 3 |
| Muscle | 1 | 0 | 1 |
| Mesentery | 2 | 0 | 2 |
| Thymus | 1 | 0 | 1 |
| Total | 7 | 18 | 25 |
