# Supplementary material for: The expression level of chicken telomerase reverse transcriptase in tumors induced by ALV-J is positively correlated with methylation and mutation of its promoter region
Source: Vet Res. 2022 Jun 23;53:49. doi: 10.1186/s13567-022-01069-2 (PMC9229480; doi:10.1186/s13567-022-01069-2)
Supplement: Supplementary file 2 — Additional file 2. Matrix of methylation levels of chTERT amplicon CG sites in LMH cells and DF-1 cells. [file 13567_2022_1069_MOESM2_ESM.doc]

**Additional file 2** Matrix of methylation levels of chTERT amplicon CG sites in LMH cells and DF-1 cells

| Position (bp) | LMH_Mock1 | LMH_Mock2 | LMH_Mock3 | LMH_  ALV_J1 | LMH_  ALV_J2 | LMH_  ALV_J3 | DF_1_  Mock1 | DF_1_ Mock2 | DF_1_ Mock3 | DF_1_  ALV_J1 | DF_1_  ALV_J2 | DF_1_  ALV_J3 |
| --- | --- | --- | --- | --- | --- | --- | --- | --- | --- | --- | --- | --- |
| -551 | 0.983 | 0.983 | 0.986 | 0.984 | 0.976 | 0.978 | 0.967 | 0.982 | 0.984 | 0.966 | 0.981 | 0.978 |
| -539 | 0.985 | 0.989 | 0.987 | 0.982 | 0.976 | 0.983 | 0.934 | 0.951 | 0.956 | 0.931 | 0.948 | 0.948 |
| -498 | 0.112 | 0.105 | 0.108 | 0.005 | 0.008 | 0.007 | 0.057 | 0.059 | 0.057 | 0.007 | 0.008 | 0.008 |
| -493 | 0.14 | 0.139 | 0.134 | 0.028 | 0.025 | 0.028 | 0.08 | 0.093 | 0.071 | 0.025 | 0.022 | 0.032 |
| -490 | 0.143 | 0.142 | 0.136 | 0.035 | 0.035 | 0.04 | 0.076 | 0.09 | 0.083 | 0.023 | 0.021 | 0.033 |
| -485 | 0.909 | 0.931 | 0.921 | 0.945 | 0.923 | 0.922 | 0.804 | 0.845 | 0.808 | 0.738 | 0.759 | 0.755 |
| -480 | 0.969 | 0.969 | 0.973 | 0.977 | 0.966 | 0.965 | 0.955 | 0.977 | 0.964 | 0.953 | 0.945 | 0.948 |
| -452 | 0.564 | 0.527 | 0.59 | 0.525 | 0.469 | 0.432 | 0.47 | 0.545 | 0.498 | 0.422 | 0.484 | 0.471 |
| -436 | 0.94 | 0.918 | 0.925 | 0.938 | 0.911 | 0.922 | 0.578 | 0.731 | 0.684 | 0.527 | 0.652 | 0.625 |
| -426 | 0.968 | 0.949 | 0.96 | 0.972 | 0.963 | 0.967 | 0.786 | 0.87 | 0.871 | 0.744 | 0.801 | 0.784 |
| -408 | 0.881 | 0.862 | 0.888 | 0.889 | 0.855 | 0.887 | 0.333 | 0.367 | 0.354 | 0.272 | 0.301 | 0.288 |
| -283 | 0.144 | 0.153 | 0.15 | 0.037 | 0.056 | 0.056 | 0.081 | 0.158 | 0.105 | 0.032 | 0.081 | 0.092 |
| -262 | 0.153 | 0.155 | 0.141 | 0.067 | 0.042 | 0.046 | 0.078 | 0.119 | 0.102 | 0.027 | 0.051 | 0.058 |
| -240 | 0.763 | 0.751 | 0.762 | 0.676 | 0.606 | 0.648 | 0.316 | 0.41 | 0.453 | 0.275 | 0.371 | 0.351 |
| -226 | 0.747 | 0.711 | 0.744 | 0.758 | 0.721 | 0.729 | 0.217 | 0.474 | 0.428 | 0.177 | 0.383 | 0.345 |
| -206 | 0.973 | 0.969 | 0.955 | 0.96 | 0.971 | 0.963 | 0.396 | 0.527 | 0.493 | 0.36 | 0.432 | 0.413 |
| -200 | 0.983 | 0.98 | 0.969 | 0.971 | 0.962 | 0.975 | 0.424 | 0.531 | 0.503 | 0.379 | 0.451 | 0.432 |
| -192 | 0.949 | 0.975 | 0.959 | 0.949 | 0.939 | 0.958 | 0.419 | 0.522 | 0.497 | 0.38 | 0.435 | 0.414 |
| -183 | 0.906 | 0.945 | 0.895 | 0.854 | 0.886 | 0.912 | 0.395 | 0.498 | 0.485 | 0.357 | 0.405 | 0.387 |
| -141 | 0.942 | 0.954 | 0.941 | 0.941 | 0.923 | 0.959 | 0.603 | 0.523 | 0.582 | 0.515 | 0.451 | 0.487 |
| -129 | 0.661 | 0.664 | 0.717 | 0.574 | 0.572 | 0.587 | 0.624 | 0.525 | 0.574 | 0.516 | 0.435 | 0.465 |
| -118 | 0.718 | 0.706 | 0.67 | 0.587 | 0.6 | 0.651 | 0.444 | 0.363 | 0.421 | 0.377 | 0.325 | 0.37 |
| -111 | 0.888 | 0.902 | 0.881 | 0.791 | 0.78 | 0.757 | 0.425 | 0.448 | 0.48 | 0.34 | 0.333 | 0.36 |
| -104 | 0.98 | 0.911 | 0.987 | 0.889 | 0.897 | 0.894 | 0.602 | 0.498 | 0.556 | 0.507 | 0.423 | 0.463 |
| -99 | 0.929 | 0.908 | 0.89 | 0.781 | 0.79 | 0.809 | 0.548 | 0.45 | 0.452 | 0.473 | 0.375 | 0.397 |
| -86 | 0.524 | 0.499 | 0.501 | 0.34 | 0.371 | 0.375 | 0.326 | 0.411 | 0.448 | 0.261 | 0.329 | 0.354 |
| -73 | 0.244 | 0.206 | 0.236 | 0.119 | 0.109 | 0.106 | 0.108 | 0.224 | 0.188 | 0.056 | 0.111 | 0.144 |
| -67 | 0.734 | 0.678 | 0.7 | 0.621 | 0.634 | 0.612 | 0.384 | 0.447 | 0.477 | 0.295 | 0.382 | 0.388 |
| -62 | 0.939 | 0.935 | 0.919 | 0.928 | 0.927 | 0.92 | 0.485 | 0.506 | 0.534 | 0.41 | 0.402 | 0.453 |
| -57 | 0.901 | 0.874 | 0.906 | 0.892 | 0.865 | 0.891 | 0.435 | 0.47 | 0.47 | 0.342 | 0.39 | 0.417 |
| -41 | 0.96 | 0.956 | 0.944 | 0.952 | 0.975 | 0.952 | 0.555 | 0.469 | 0.491 | 0.446 | 0.38 | 0.414 |
| -36 | 0.941 | 0.95 | 0.949 | 0.947 | 0.957 | 0.954 | 0.434 | 0.42 | 0.475 | 0.367 | 0.337 | 0.376 |
| -27 | 0.944 | 0.952 | 0.949 | 0.941 | 0.955 | 0.946 | 0.606 | 0.5 | 0.558 | 0.49 | 0.42 | 0.457 |
| -20 | 0.675 | 0.656 | 0.723 | 0.606 | 0.597 | 0.586 | 0.441 | 0.509 | 0.521 | 0.483 | 0.407 | 0.425 |
| -14 | 0.938 | 0.817 | 0.981 | 0.719 | 0.752 | 0.747 | 0.578 | 0.52 | 0.484 | 0.512 | 0.341 | 0.418 |
| -9 | 0.83 | 0.799 | 0.865 | 0.522 | 0.553 | 0.672 | 0.55 | 0.428 | 0.424 | 0.25 | 0.232 | 0.317 |
| -5 | 0.705 | 0.688 | 0.656 | 0.494 | 0.511 | 0.516 | 1 | 0.436 | 0.386 | 0.5 | 0.349 | 0.441 |
| 7 | 0.991 | 0.995 | 0.987 | 0.988 | 0.985 | 0.985 | 1 | 0.481 | 0.517 | 1 | 0.446 | 0.48 |
| 9 | 0.988 | 0.988 | 0.992 | 0.992 | 0.992 | 0.983 | 0.8 | 0.527 | 0.567 | 1 | 0.449 | 0.475 |
| 20 | 0.754 | 0.721 | 0.715 | 0.758 | 0.735 | 0.737 | 0.54 | 0.489 | 0.539 | 0.458 | 0.425 | 0.46 |
| 27 | 0.982 | 0.968 | 0.978 | 0.978 | 0.972 | 0.973 | 0.618 | 0.536 | 0.565 | 0.52 | 0.434 | 0.473 |
| 34 | 0.986 | 0.972 | 0.977 | 0.979 | 0.98 | 0.978 | 0.614 | 0.532 | 0.571 | 0.524 | 0.445 | 0.475 |
| 37 | 0.978 | 0.968 | 0.974 | 0.969 | 0.971 | 0.971 | 0.625 | 0.532 | 0.565 | 0.513 | 0.436 | 0.477 |
| 43 | 0.968 | 0.957 | 0.968 | 0.959 | 0.97 | 0.963 | 0.61 | 0.519 | 0.573 | 0.514 | 0.444 | 0.479 |
| 53 | 0.715 | 0.711 | 0.698 | 0.598 | 0.6 | 0.618 | 0.587 | 0.528 | 0.564 | 0.491 | 0.433 | 0.473 |
| 55 | 0.691 | 0.696 | 0.683 | 0.617 | 0.583 | 0.615 | 0.519 | 0.469 | 0.521 | 0.464 | 0.39 | 0.435 |
| 69 | 0.957 | 0.975 | 0.971 | 0.969 | 0.972 | 0.968 | 0.612 | 0.523 | 0.568 | 0.51 | 0.434 | 0.478 |
| 72 | 0.969 | 0.985 | 0.98 | 0.978 | 0.969 | 0.98 | 0.612 | 0.533 | 0.573 | 0.506 | 0.443 | 0.481 |
| 74 | 0.957 | 0.968 | 0.955 | 0.939 | 0.948 | 0.946 | 0.561 | 0.516 | 0.571 | 0.454 | 0.437 | 0.467 |
| 84 | 0.971 | 0.971 | 0.98 | 0.979 | 0.969 | 0.972 | 0.606 | 0.535 | 0.575 | 0.514 | 0.446 | 0.475 |
| 86 | 0.976 | 0.989 | 0.976 | 0.969 | 0.98 | 0.978 | 0.617 | 0.536 | 0.581 | 0.519 | 0.446 | 0.471 |
| 91 | 0.982 | 0.977 | 0.983 | 0.971 | 0.973 | 0.975 | 0.613 | 0.531 | 0.564 | 0.505 | 0.436 | 0.467 |
| 102 | 0.968 | 0.98 | 0.984 | 0.982 | 0.969 | 0.971 | 0.602 | 0.527 | 0.546 | 0.499 | 0.441 | 0.473 |
| 105 | 0.945 | 0.937 | 0.955 | 0.941 | 0.942 | 0.93 | 0.56 | 0.505 | 0.557 | 0.481 | 0.407 | 0.447 |
| 113 | 0.951 | 0.934 | 0.931 | 0.938 | 0.94 | 0.918 | 0.457 | 0.469 | 0.515 | 0.385 | 0.405 | 0.438 |
| 116 | 0.978 | 0.985 | 0.986 | 0.982 | 0.982 | 0.982 | 0.605 | 0.537 | 0.576 | 0.502 | 0.437 | 0.477 |
| 129 | 0.969 | 0.989 | 0.991 | 0.988 | 0.983 | 0.98 | 0.617 | 0.533 | 0.559 | 0.509 | 0.444 | 0.478 |
| 133 | 0.979 | 0.978 | 0.973 | 0.984 | 0.983 | 0.977 | 0.621 | 0.524 | 0.568 | 0.524 | 0.445 | 0.478 |
| 136 | 0.989 | 0.979 | 0.984 | 0.973 | 0.979 | 0.975 | 0.61 | 0.53 | 0.574 | 0.514 | 0.438 | 0.479 |
| 294 | 0.973 | 0.985 | 0.974 | 0.971 | 0.978 | 0.971 | 0.488 | 0.592 | 0.585 | 0.395 | 0.533 | 0.528 |
| 298 | 0.933 | 0.959 | 0.937 | 0.951 | 0.935 | 0.944 | 0.437 | 0.538 | 0.574 | 0.336 | 0.493 | 0.495 |
| 309 | 0.984 | 0.975 | 0.982 | 0.983 | 0.987 | 0.97 | 0.486 | 0.589 | 0.596 | 0.395 | 0.529 | 0.524 |
| 313 | 0.984 | 0.985 | 0.989 | 0.98 | 0.989 | 0.98 | 0.472 | 0.56 | 0.596 | 0.381 | 0.526 | 0.506 |
| 315 | 0.975 | 0.983 | 0.981 | 0.982 | 0.975 | 0.976 | 0.466 | 0.578 | 0.594 | 0.379 | 0.522 | 0.515 |
| 319 | 0.982 | 0.968 | 0.987 | 0.983 | 0.983 | 0.981 | 0.467 | 0.591 | 0.598 | 0.386 | 0.525 | 0.523 |
| 324 | 0.982 | 0.977 | 0.991 | 0.986 | 0.977 | 0.984 | 0.468 | 0.582 | 0.582 | 0.383 | 0.508 | 0.515 |
| 326 | 0.988 | 0.987 | 0.989 | 0.977 | 0.975 | 0.983 | 0.462 | 0.559 | 0.582 | 0.383 | 0.518 | 0.524 |
| 330 | 0.918 | 0.918 | 0.908 | 0.888 | 0.865 | 0.898 | 0.445 | 0.559 | 0.563 | 0.352 | 0.503 | 0.498 |
| 342 | 0.953 | 0.963 | 0.96 | 0.963 | 0.952 | 0.944 | 0.448 | 0.56 | 0.568 | 0.363 | 0.503 | 0.501 |
| 354 | 0.979 | 0.962 | 0.967 | 0.971 | 0.969 | 0.975 | 0.459 | 0.563 | 0.584 | 0.373 | 0.514 | 0.517 |
| 363 | 0.826 | 0.869 | 0.84 | 0.869 | 0.87 | 0.852 | 0.458 | 0.559 | 0.572 | 0.369 | 0.498 | 0.505 |
| 382 | 0.964 | 0.961 | 0.961 | 0.945 | 0.96 | 0.958 | 0.461 | 0.565 | 0.57 | 0.367 | 0.5 | 0.492 |
| 384 | 0.927 | 0.922 | 0.919 | 0.909 | 0.925 | 0.936 | 0.459 | 0.563 | 0.568 | 0.372 | 0.506 | 0.496 |
| 394 | 0.946 | 0.941 | 0.953 | 0.936 | 0.96 | 0.969 | 0.43 | 0.554 | 0.55 | 0.345 | 0.471 | 0.479 |
| 398 | 0.943 | 0.949 | 0.938 | 0.946 | 0.971 | 0.961 | 0.456 | 0.553 | 0.562 | 0.365 | 0.486 | 0.48 |
| 407 | 0.483 | 0.489 | 0.487 | 0.235 | 0.249 | 0.399 | 0.241 | 0.319 | 0.293 | 0.174 | 0.227 | 0.235 |
| 413 | 0.972 | 0.973 | 0.959 | 0.797 | 0.806 | 0.834 | 0.377 | 0.513 | 0.539 | 0.287 | 0.463 | 0.464 |
| 421 | 0.298 | 0.316 | 0.322 | 0.126 | 0.13 | 0.176 | 0.36 | 0.527 | 0.528 | 0.268 | 0.451 | 0.454 |
| 425 | 0.302 | 0.312 | 0.314 | 0.125 | 0.129 | 0.182 | 0.397 | 0.561 | 0.533 | 0.309 | 0.451 | 0.464 |
| 427 | 0.96 | 0.953 | 0.961 | 0.945 | 0.955 | 0.945 | 0.418 | 0.55 | 0.542 | 0.328 | 0.485 | 0.474 |
| 432 | 0.206 | 0.221 | 0.212 | 0.092 | 0.093 | 0.101 | 0.16 | 0.295 | 0.243 | 0.091 | 0.199 | 0.196 |
| 437 | 0.896 | 0.902 | 0.905 | 0.894 | 0.915 | 0.828 | 0.41 | 0.561 | 0.542 | 0.323 | 0.485 | 0.485 |
| 448 | 0.888 | 0.903 | 0.893 | 0.866 | 0.866 | 0.793 | 0.377 | 0.539 | 0.541 | 0.304 | 0.433 | 0.448 |
| 454 | 0.931 | 0.953 | 0.974 | 0.848 | 0.86 | 0.869 | 0.394 | 0.576 | 0.563 | 0.304 | 0.484 | 0.48 |
| 470 | 0.974 | 0.95 | 0.991 | 0.85 | 0.855 | 0.859 | 0.437 | 0.581 | 0.574 | 0.344 | 0.504 | 0.511 |
| 473 | 0.301 | 0.318 | 0.326 | 0.133 | 0.13 | 0.186 | 0.419 | 0.572 | 0.544 | 0.327 | 0.49 | 0.49 |
| 478 | 0.982 | 0.985 | 0.988 | 0.984 | 0.978 | 0.969 | 0.427 | 0.565 | 0.552 | 0.337 | 0.49 | 0.493 |
| 487 | 0.97 | 0.967 | 0.988 | 0.979 | 0.988 | 0.975 | 0.423 | 0.588 | 0.56 | 0.328 | 0.498 | 0.488 |
| 491 | 0.949 | 0.974 | 0.967 | 0.961 | 0.953 | 0.945 | 0.402 | 0.559 | 0.55 | 0.312 | 0.49 | 0.514 |
| 495 | 0.932 | 0.971 | 0.958 | 0.944 | 0.955 | 0.963 | 0.428 | 0.575 | 0.566 | 0.334 | 0.507 | 0.494 |
| 498 | 0.716 | 0.69 | 0.686 | 0.436 | 0.482 | 0.718 | 0.428 | 0.582 | 0.568 | 0.34 | 0.505 | 0.508 |
| 631 | 0.982 | 0.986 | 0.985 | 0.977 | 0.975 | 0.979 | 0.974 | 0.993 | 0.988 | 0.965 | 0.983 | 0.993 |
| 639 | 0.982 | 0.98 | 0.982 | 0.976 | 0.976 | 0.976 | 0.965 | 0.991 | 0.986 | 0.96 | 0.984 | 0.984 |
| 642 | 0.985 | 0.979 | 0.991 | 0.986 | 0.984 | 0.983 | 0.98 | 0.995 | 0.984 | 0.98 | 0.994 | 0.986 |
| 644 | 0.986 | 0.986 | 0.991 | 0.983 | 0.984 | 0.984 | 0.979 | 0.981 | 0.987 | 0.971 | 0.983 | 0.98 |
| 646 | 0.985 | 0.984 | 0.986 | 0.985 | 0.977 | 0.982 | 0.984 | 0.982 | 0.982 | 0.981 | 0.983 | 0.979 |
| 651 | 0.973 | 0.972 | 0.973 | 0.966 | 0.967 | 0.967 | 0.918 | 0.942 | 0.973 | 0.918 | 0.949 | 0.948 |
| 660 | 0.989 | 0.983 | 0.992 | 0.986 | 0.982 | 0.988 | 0.977 | 0.997 | 0.979 | 0.97 | 0.989 | 0.979 |
| 669 | 0.985 | 0.977 | 0.985 | 0.981 | 0.983 | 1 | 0.964 | 0.987 | 0.978 | 0.967 | 0.965 | 0.985 |
| 673 | 0.975 | 0.974 | 0.969 | 0.972 | 0.971 | 0.964 | 0.949 | 0.961 | 0.967 | 1 | 0.968 | 0.968 |
| 707 | 0.953 | 0.948 | 0.953 | 0.946 | 0.948 | 0.941 | 0.956 | 0.967 | 0.977 | 0.95 | 0.979 | 0.985 |
| 728 | 0.306 | 0.328 | 0.324 | 0.208 | 0.196 | 0.226 | 0.351 | 0.5 | NA | 0.34 | 0.516 | 0.518 |
| 737 | 0.75 | 0.799 | 0.793 | 0.667 | 0.664 | 0.668 | 0.636 | 0.781 | 0.793 | 0.616 | 0.789 | 0.809 |
| 746 | 0.973 | 0.976 | 0.981 | 0.976 | 0.973 | 0.976 | 0.976 | 0.979 | 0.982 | 0.976 | 0.982 | 0.985 |
| 752 | 0.987 | 0.973 | 0.978 | 0.976 | 0.977 | 0.984 | 0.971 | 0.979 | 0.986 | 0.985 | 0.987 | 0.982 |
| 768 | 0.955 | 0.946 | 0.942 | 0.947 | 0.945 | 0.943 | 0.96 | 0.973 | 0.973 | 0.961 | 0.983 | 0.971 |
| 782 | 0.939 | 0.932 | 0.935 | 0.931 | 0.941 | 0.942 | 0.905 | 0.966 | 0.924 | 0.926 | 0.944 | 0.952 |
| 811 | 0.778 | 0.754 | 0.76 | 0.757 | 0.749 | 0.752 | 0.598 | 0.843 | 0.817 | 0.63 | 0.823 | 0.855 |
| 836 | 0.981 | 0.978 | 0.973 | 0.974 | 0.973 | 0.963 | 0.962 | 0.991 | 0.972 | 0.958 | 0.976 | 0.986 |
| 844 | 0.787 | 0.793 | 0.795 | 0.805 | 0.792 | 0.775 | 0.909 | 0.933 | 0.906 | 0.878 | 0.919 | 0.918 |
