# Supplementary material for: The expression level of chicken telomerase reverse transcriptase in tumors induced by ALV-J is positively correlated with methylation and mutation of its promoter region
Source: Vet Res. 2022 Jun 23;53:49. doi: 10.1186/s13567-022-01069-2 (PMC9229480; doi:10.1186/s13567-022-01069-2)
Supplement: Supplementary file 3 — Additional file 3. Matrix of methylation levels of chTERT amplicon CG sites in ALV-J tumor, tumor-adjacent and normal tissues. [file 13567_2022_1069_MOESM3_ESM.doc]

**Additional file 3 Matrix of methylation levels of chTERT amplicon CG sites in ALV-J** tumor, tumor-adjacent and normal tissues

| Position  (bp) | Kidney_N | Kidney_TA | Kidney_T1 | Kidney_T2 | Liver_N | Liver_T1 | Liver_T2 | Liver_T3 | Liver_TA | Mesen-tery_N | Mesen-tery_T | Mesen-tery_TA | Spleen  _T1 | Spleen  _T2 | Spleen  _TA | Spleen  _N |
| --- | --- | --- | --- | --- | --- | --- | --- | --- | --- | --- | --- | --- | --- | --- | --- | --- |
| -551 | 0.968 | 0.956 | 0.952 | 0.972 | 0.968 | 0.956 | 0.958 | 0.962 | 0.931 | 0.945 | 0.97 | 0.968 | 0.958 | 0.965 | NA | 0.976 |
| -539 | 0.912 | 0.862 | 0.918 | 0.854 | 0.904 | 0.895 | 0.939 | 0.904 | 1 | 0.869 | 0.935 | 0.935 | 0.936 | 0.937 | NA | 0.944 |
| -498 | 0.007 | 0.01 | 0.01 | 0.009 | 0.009 | 0.009 | 0.009 | 0.009 | 0 | 0.006 | 0.014 | 0.012 | 0.008 | 0.008 | NA | 0.005 |
| -493 | 0.019 | 0.014 | 0.024 | 0.012 | 0.019 | 0.026 | 0.033 | 0.012 | 0 | 0.016 | 0.029 | 0.023 | 0.024 | 0.022 | NA | 0.017 |
| -490 | 0.021 | 0.013 | 0.041 | 0.018 | 0.021 | 0.021 | 0.035 | 0.016 | 0 | 0.021 | 0.045 | 0.024 | 0.022 | 0.021 | NA | 0.014 |
| -485 | 0.326 | 0.255 | 0.598 | 0.455 | 0.457 | 0.593 | 0.701 | 0.299 | 0.72 | 0.449 | 0.604 | 0.604 | 0.645 | 0.592 | NA | 0.471 |
| -480 | 0.598 | 0.6 | 0.843 | 0.738 | 0.868 | 0.837 | 0.893 | 0.81 | 0.815 | 0.74 | 0.828 | 0.843 | 0.839 | 0.826 | NA | 0.753 |
| -452 | 0.272 | 0.195 | 0.615 | 0.47 | 0.483 | 0.613 | 0.746 | 0.601 | 0.731 | 0.404 | 0.607 | 0.674 | 0.673 | 0.66 | NA | 0.433 |
| -436 | 0.277 | 0.233 | 0.671 | 0.382 | 0.512 | 0.639 | 0.809 | 0.657 | 0.577 | 0.468 | 0.679 | 0.691 | 0.74 | 0.677 | NA | 0.365 |
| -426 | 0.36 | 0.378 | 0.808 | 0.584 | 0.629 | 0.755 | 0.905 | 0.784 | 0.846 | 0.69 | 0.809 | 0.823 | 0.867 | 0.808 | NA | 0.604 |
| -408 | 0.215 | 0.209 | 0.646 | 0.298 | 0.246 | 0.524 | 0.751 | 0.577 | 0.615 | 0.442 | 0.663 | 0.576 | 0.574 | 0.605 | NA | 0.205 |
| -283 | 0.011 | 0.012 | 0.031 | 0.011 | 0.017 | 0.024 | 0.018 | 0.015 | 0.026 | 0.015 | 0.046 | 0.015 | 0.012 | 0.017 | 0.013 | 0.01 |
| -262 | 0.014 | 0.013 | 0.09 | 0.013 | 0.017 | 0.012 | 0.018 | 0.011 | 0.019 | 0.011 | 0.139 | 0.016 | 0.014 | 0.011 | 0.018 | 0.008 |
| -240 | 0.028 | 0.024 | 0.177 | 0.025 | 0.041 | 0.027 | 0.121 | 0.065 | 0.038 | 0.035 | 0.239 | 0.04 | 0.042 | 0.031 | 0.034 | 0.018 |
| -226 | 0.118 | 0.15 | 0.336 | 0.052 | 0.153 | 0.135 | 0.316 | 0.223 | 0.084 | 0.247 | 0.366 | 0.211 | 0.14 | 0.154 | 0.092 | 0.049 |
| -206 | 0.105 | 0.138 | 0.363 | 0.053 | 0.108 | 0.114 | 0.285 | 0.197 | 0.037 | 0.236 | 0.382 | 0.162 | 0.12 | 0.11 | 0.041 | 0.053 |
| -200 | 0.118 | 0.161 | 0.394 | 0.058 | 0.122 | 0.121 | 0.369 | 0.231 | 0.004 | 0.305 | 0.408 | 0.174 | 0.157 | 0.128 | 0.004 | 0.058 |
| -192 | 0.107 | 0.163 | 0.401 | 0.059 | 0.097 | 0.114 | 0.329 | 0.214 | 0.031 | 0.306 | 0.407 | 0.141 | 0.148 | 0.11 | 0.035 | 0.056 |
| -183 | 0.026 | 0.041 | 0.239 | 0.014 | 0.006 | 0.037 | 0.098 | 0.07 | 0.003 | 0.075 | 0.391 | 0.004 | 0.03 | 0.025 | 0.003 | 0.009 |
| -141 | 0.038 | 0.02 | 0.192 | 0.018 | 0.04 | 0.032 | 0.066 | 0.026 | 0.033 | 0.105 | 0.303 | 0.025 | 0.026 | 0.018 | 0.02 | 0.026 |
| -129 | 0.011 | 0 | 0.113 | 0.011 | 0.01 | 0.021 | 0.021 | 0.012 | 0.01 | 0 | 0.157 | 0.011 | 0.017 | 0.004 | 0.005 | 0.009 |
| -118 | 0.01 | 0.01 | 0.02 | 0.008 | 0.006 | 0.014 | 0.016 | 0.007 | 0.01 | 0.018 | 0.017 | 0.006 | 0.008 | 0.011 | 0.006 | 0.008 |
| -111 | 0.014 | 0.01 | 0 | 0.01 | 0.007 | 0.016 | 0.015 | 0.008 | 0.004 | 0.027 | 0 | 0.011 | 0.01 | 0.014 | 0.009 | 0.011 |
| -104 | 0.014 | 0 | 0.033 | 0.016 | 0.016 | 0.013 | 0.016 | 0.003 | 0.018 | 0.009 | 0.013 | 0.01 | 0.017 | 0.011 | 0.009 | 0.01 |
| -99 | 0.004 | 0.021 | 0.013 | 0.009 | 0.009 | 0.009 | 0.019 | 0.015 | 0.009 | 0.019 | 0.007 | 0.006 | 0.008 | 0.013 | 0.008 | 0.008 |
| -86 | 0.01 | 0 | 0 | 0.006 | 0.004 | 0.01 | 0.007 | 0.009 | 0.007 | 0.018 | 0.027 | 0.006 | 0.009 | 0.012 | 0.005 | 0.008 |
| -73 | 0.007 | 0 | 0.013 | 0.003 | 0.011 | 0.005 | 0.01 | 0.008 | 0.009 | 0.009 | 0.01 | 0.009 | 0.012 | 0.012 | 0.004 | 0.011 |
| -67 | 0.009 | 0.02 | 0.06 | 0.003 | 0.015 | 0.008 | 0.022 | 0.014 | 0.01 | 0.045 | 0.081 | 0.018 | 0.012 | 0.008 | 0.009 | 0.009 |
| -62 | 0.018 | 0 | 0.059 | 0.006 | 0.015 | 0.011 | 0.01 | 0.023 | 0.013 | 0.037 | 0.044 | 0.011 | 0.011 | 0.009 | 0.007 | 0.003 |
| -57 | 0.008 | 0.01 | 0.053 | 0.011 | 0.015 | 0.012 | 0.019 | 0.015 | 0.008 | 0.018 | 0.061 | 0.014 | 0.009 | 0.013 | 0.01 | 0.011 |
| -41 | 0.017 | 0 | 0.069 | 0.021 | 0.015 | 0.019 | 0.027 | 0.012 | 0.011 | NA | 0.1 | 0.015 | 0.02 | 0.008 | 0.008 | 0.014 |
| -36 | 0.011 | 0.02 | 0.007 | 0.006 | 0.009 | 0.013 | 0.013 | 0.015 | 0.004 | 0.018 | 0.038 | 0.006 | 0.008 | 0.008 | 0.006 | 0.007 |
| -27 | 0.019 | 0 | 0.041 | 0.013 | 0.013 | 0.012 | 0.011 | 0.012 | 0.005 | 0 | 0.056 | 0.013 | 0.011 | 0.008 | 0.007 | 0.011 |
| -20 | 0 | 0 | 0 | 0 | 0 | 0 | 0.014 | 0 | 0 | 0 | 0 | 0 | 0 | 0 | 0.017 | 0 |
| -14 | 0 | 0 | 0 | 0 | 0 | 0 | 0 | 0 | 0 | NA | 0 | 0 | 0 | 0 | 0 | 0 |
| -9 | 0 | NA | 0 | 0 | 0 | 0.034 | 0 | 0 | 0 | NA | NA | 0 | 0 | 0 | 0 | 0 |
| -5 | 0 | NA | 0 | 0 | 0 | 0.077 | 0 | 0 | 0 | NA | NA | 0 | 0 | 0 | 0 | 0 |
| 7 | 0 | NA | NA | 0 | 0.077 | 0 | 0 | 0 | 0 | NA | 0.25 | 0 | 0 | 0 | 0 | 0 |
| 9 | 0 | NA | NA | 0 | 0 | 0 | 0 | 0 | 0 | 0 | 0 | 0 | 0 | 0 | 0 | 0 |
| 20 | 0.008 | 0.019 | 0.031 | 0.013 | 0.012 | 0.007 | 0.017 | 0.008 | 0.01 | 0.008 | 0.029 | 0.009 | 0.006 | 0.012 | 0.006 | 0.01 |
| 27 | 0.01 | 0 | 0.013 | 0.007 | 0.008 | 0.01 | 0.014 | 0.01 | 0.006 | 0.008 | 0.036 | 0.009 | 0.007 | 0.005 | 0.008 | 0.002 |
| 34 | 0.011 | 0 | 0.062 | 0.009 | 0.01 | 0.011 | 0.017 | 0.008 | 0.008 | 0.026 | 0.089 | 0.008 | 0.012 | 0.007 | 0.011 | 0.01 |
| 37 | 0.006 | 0.009 | 0.037 | 0.006 | 0.011 | 0.005 | 0.007 | 0.005 | 0.005 | 0 | 0.045 | 0.007 | 0.01 | 0.008 | 0.011 | 0.01 |
| 43 | 0.021 | 0.027 | 0.129 | 0.022 | 0.019 | 0.019 | 0.035 | 0.022 | 0.017 | 0.048 | 0.126 | 0.017 | 0.018 | 0.015 | 0.02 | 0.017 |
| 53 | 0.008 | 0 | 0.031 | 0.007 | 0.011 | 0.012 | 0.014 | 0.011 | 0.014 | 0 | 0.047 | 0.009 | 0.016 | 0.004 | 0.011 | 0.01 |
| 55 | 0.01 | 0.028 | 0.031 | 0.008 | 0.007 | 0.011 | 0.014 | 0.007 | 0.006 | 0 | 0.025 | 0.005 | 0.005 | 0.012 | 0.006 | 0.008 |
| 69 | 0.006 | 0.018 | 0.055 | 0.017 | 0.011 | 0.012 | 0.014 | 0.012 | 0.005 | 0.008 | 0.064 | 0.01 | 0.014 | 0.009 | 0.015 | 0.008 |
| 72 | 0.014 | 0 | 0.031 | 0.015 | 0.014 | 0.017 | 0.019 | 0.012 | 0.015 | 0 | 0.012 | 0.008 | 0.018 | 0.017 | 0.013 | 0.012 |
| 74 | 0.014 | 0.027 | 0.018 | 0.005 | 0.004 | 0.006 | 0.016 | 0.008 | 0.005 | 0.008 | 0.027 | 0.007 | 0.008 | 0.012 | 0.008 | 0.005 |
| 84 | 0.008 | 0.018 | 0.025 | 0.009 | 0.017 | 0.006 | 0.011 | 0.014 | 0.013 | 0 | 0.07 | 0.006 | 0.006 | 0.006 | 0.008 | 0.003 |
| 86 | 0.015 | 0.009 | 0.012 | 0.007 | 0.011 | 0.008 | 0.007 | 0.009 | 0.009 | 0.008 | 0.052 | 0.014 | 0.011 | 0.01 | 0.008 | 0.009 |
| 91 | 0.009 | 0.009 | 0.018 | 0.005 | 0.01 | 0.003 | 0.006 | 0.007 | 0.007 | 0.038 | 0.052 | 0.01 | 0.005 | 0.009 | 0.005 | 0.008 |
| 102 | 0.013 | 0.009 | 0.091 | 0.016 | 0.019 | 0.016 | 0.019 | 0.014 | 0.021 | 0.008 | 0.094 | 0.02 | 0.022 | 0.012 | 0.016 | 0.005 |
| 105 | 0.007 | 0.009 | 0.018 | 0.022 | 0.012 | 0.021 | 0.02 | 0.014 | 0.014 | 0.015 | 0.036 | 0.018 | 0.01 | 0.011 | 0.01 | 0.013 |
| 113 | 0.014 | 0.018 | 0.061 | 0.018 | 0.014 | 0.019 | 0.023 | 0.019 | 0.018 | 0.015 | 0.076 | 0.023 | 0.014 | 0.011 | 0.012 | 0.011 |
| 116 | 0.012 | 0.062 | 0.097 | 0.022 | 0.021 | 0.035 | 0.017 | 0.019 | 0.018 | 0 | 0.054 | 0.022 | 0.024 | 0.016 | 0.018 | 0.013 |
| 129 | 0.021 | 0.027 | 0.079 | 0.015 | 0.031 | 0.022 | 0.02 | 0.013 | 0.02 | 0.008 | 0.154 | 0.018 | 0.025 | 0.008 | 0.012 | 0.009 |
| 133 | 0.023 | 0.009 | 0.037 | 0.013 | 0.024 | 0.018 | 0.032 | 0.019 | 0.024 | 0.016 | 0.112 | 0.017 | 0.023 | 0.017 | 0.02 | 0.012 |
| 136 | 0.008 | 0.018 | 0.061 | 0.01 | 0.016 | 0.018 | 0.016 | 0.018 | 0.011 | 0.008 | 0.094 | 0.017 | 0.011 | 0.013 | 0.009 | 0.006 |
| 294 | 0.03 | 0.097 | 0.061 | 0.106 | 0.14 | 0.055 | 0.045 | 0.028 | 0.057 | 0.04 | 0.093 | 0.039 | 0.033 | 0.021 | 0.035 | 0.012 |
| 298 | 0.023 | 0.053 | 0.047 | 0.053 | 0.072 | 0.044 | 0.042 | 0.025 | 0.031 | 0.023 | 0.075 | 0.028 | 0.026 | 0.029 | 0.026 | 0.014 |
| 309 | 0.051 | 0.096 | 0.07 | 0.114 | 0.214 | 0.079 | 0.062 | 0.064 | 0.059 | 0.034 | 0.11 | 0.072 | 0.046 | 0.036 | 0.043 | 0.018 |
| 313 | 0.031 | 0.055 | 0.045 | 0.074 | 0.104 | 0.064 | 0.043 | 0.034 | 0.038 | 0.021 | 0.056 | 0.048 | 0.025 | 0.02 | 0.033 | 0.015 |
| 315 | 0.04 | 0.078 | 0.056 | 0.099 | 0.14 | 0.065 | 0.036 | 0.045 | 0.056 | 0.027 | 0.071 | 0.048 | 0.039 | 0.023 | 0.044 | 0.018 |
| 319 | 0.037 | 0.074 | 0.062 | 0.103 | 0.146 | 0.074 | 0.059 | 0.044 | 0.058 | 0.033 | 0.079 | 0.063 | 0.049 | 0.037 | 0.041 | 0.018 |
| 324 | 0.019 | 0.047 | 0.036 | 0.052 | 0.076 | 0.027 | 0.031 | 0.026 | 0.037 | 0.015 | 0.046 | 0.028 | 0.024 | 0.037 | 0.024 | 0.011 |
| 326 | 0.026 | 0.056 | 0.043 | 0.061 | 0.078 | 0.033 | 0.034 | 0.029 | 0.028 | 0.016 | 0.071 | 0.039 | 0.024 | 0.028 | 0.028 | 0.015 |
| 330 | 0.013 | 0.021 | 0.023 | 0.029 | 0.031 | 0.017 | 0.016 | 0.014 | 0.014 | 0.012 | 0.028 | 0.009 | 0.011 | 0.005 | 0.016 | 0.007 |
| 342 | 0.02 | 0.016 | 0.029 | 0.032 | 0.016 | 0.012 | 0.013 | 0.012 | 0.012 | 0.01 | 0.045 | 0.013 | 0.015 | 0.01 | 0.012 | 0.006 |
| 354 | 0.025 | 0.029 | 0.042 | 0.028 | 0.027 | 0.028 | 0.025 | 0.017 | 0.019 | 0.014 | 0.06 | 0.017 | 0.015 | 0.017 | 0.015 | 0.007 |
| 363 | 0.014 | 0.021 | 0.023 | 0.022 | 0.02 | 0.013 | 0.029 | 0.01 | 0.012 | 0.014 | 0.03 | 0.005 | 0.014 | 0.011 | 0.013 | 0.004 |
| 382 | 0.017 | 0.039 | 0.041 | 0.047 | 0.064 | 0.02 | 0.048 | 0.025 | 0.025 | 0.015 | 0.054 | 0.028 | 0.023 | 0.026 | 0.019 | 0.011 |
| 384 | 0.034 | 0.05 | 0.051 | 0.057 | 0.074 | 0.043 | 0.059 | 0.044 | 0.027 | 0.024 | 0.076 | 0.037 | 0.034 | 0.016 | 0.021 | 0.013 |
| 394 | 0.018 | 0.023 | 0.119 | 0.028 | 0.047 | 0.032 | 0.09 | 0.026 | 0.013 | 0.01 | 0.217 | 0.015 | 0.03 | 0.021 | 0.009 | 0.009 |
| 398 | 0.02 | 0.038 | 0.056 | 0.028 | 0.054 | 0.028 | 0.04 | 0.02 | 0.034 | 0.022 | 0.078 | 0.014 | 0.027 | 0.025 | 0.022 | 0.014 |
| 407 | 0.008 | 0.014 | 0.022 | 0.012 | 0.02 | 0.012 | 0.027 | 0.011 | 0.01 | 0.008 | 0.034 | 0.006 | 0.015 | 0.014 | 0.011 | 0.005 |
| 413 | 0.028 | 0.04 | 0.043 | 0.051 | 0.099 | 0.031 | 0.03 | 0.024 | 0.036 | 0.026 | 0.082 | 0.039 | 0.024 | 0.033 | 0.024 | 0.021 |
| 421 | 0.039 | 0.065 | 0.063 | 0.043 | 0.139 | 0.035 | 0.032 | 0.029 | 0.057 | 0.029 | 0.095 | 0.044 | 0.024 | 0.043 | 0.036 | 0.011 |
| 425 | 0.018 | 0.024 | 0.027 | 0.011 | 0.051 | 0.015 | 0.022 | 0.012 | 0.024 | 0.015 | 0.032 | 0.016 | 0.02 | 0.009 | 0.014 | 0.009 |
| 427 | 0.013 | 0.016 | 0.022 | 0.015 | 0.037 | 0.01 | 0.013 | 0.014 | 0.012 | 0.007 | 0.021 | 0.016 | 0.012 | 0.01 | 0.007 | 0.009 |
| 432 | 0.018 | 0.014 | 0.013 | 0.013 | 0.033 | 0.006 | 0.008 | 0.013 | 0.02 | 0.01 | 0.018 | 0.011 | 0.014 | 0.015 | 0.012 | 0.012 |
| 437 | 0.026 | 0.031 | 0.024 | 0.022 | 0.007 | 0.018 | 0.026 | 0.026 | 0.041 | 0.019 | 0.039 | 0.007 | 0.02 | 0.015 | 0.019 | 0.007 |
| 448 | 0.032 | 0.058 | 0.036 | 0.025 | 0.106 | 0.018 | 0.024 | 0.018 | 0.071 | 0.025 | 0.037 | 0.016 | 0.02 | 0.014 | 0.031 | 0.011 |
| 454 | 0.083 | 0.122 | 0.085 | 0.165 | 0.381 | 0.118 | 0.078 | 0.079 | 0.132 | 0.053 | 0.123 | 0.08 | 0.071 | 0.044 | 0.055 | 0.021 |
| 470 | 0.046 | 0.097 | 0.089 | 0.112 | 0.24 | 0.078 | 0.086 | 0.074 | 0.094 | 0.045 | 0.099 | 0.047 | 0.061 | 0.026 | 0.051 | 0.025 |
| 473 | 0.106 | 0.196 | 0.142 | 0.204 | 0.441 | 0.163 | 0.112 | 0.138 | 0.229 | 0.074 | 0.162 | 0.089 | 0.094 | 0.072 | 0.093 | 0.016 |
| 478 | 0.044 | 0.102 | 0.06 | 0.092 | 0.203 | 0.096 | 0.074 | 0.079 | 0.1 | 0.044 | 0.083 | 0.048 | 0.049 | 0.049 | 0.058 | 0.018 |
| 487 | 0.071 | 0.156 | 0.097 | 0.192 | 0.382 | 0.17 | 0.186 | 0.134 | 0.175 | 0.059 | 0.143 | 0.068 | 0.08 | 0.068 | 0.085 | 0.021 |
| 491 | 0.108 | 0.191 | 0.135 | 0.242 | 0.451 | 0.188 | 0.144 | 0.15 | 0.209 | 0.079 | 0.136 | 0.122 | 0.084 | 0.087 | 0.087 | 0.033 |
| 495 | 0.107 | 0.208 | 0.131 | 0.263 | 0.473 | 0.19 | 0.161 | 0.148 | 0.214 | 0.096 | 0.154 | 0.122 | 0.102 | 0.084 | 0.117 | 0.03 |
| 498 | 0.028 | 0.011 | 0.035 | 0.201 | 0.431 | 0.164 | 0.17 | 0.128 | 0.016 | 0.011 | 0.061 | 0.092 | 0.086 | 0.07 | 0.017 | 0.022 |
| 631 | 0.363 | 0.453 | 0.433 | 0.584 | 0.722 | 0.395 | 0.395 | 0.405 | 0.561 | 0.295 | 0.587 | 0.235 | 0.309 | 0.223 | 0.269 | 0.146 |
| 639 | 0.376 | 0.474 | 0.458 | 0.602 | 0.738 | 0.448 | 0.576 | 0.511 | 0.651 | 0.327 | 0.619 | 0.258 | 0.347 | 0.245 | 0.293 | 0.141 |
| 642 | 0.25 | 0.34 | 0.364 | 0.424 | 0.585 | 0.328 | 0.323 | 0.334 | 0.42 | 0.203 | 0.498 | 0.18 | 0.213 | 0.172 | 0.205 | 0.098 |
| 644 | 0.194 | 0.278 | 0.305 | 0.348 | 0.522 | 0.252 | 0.291 | 0.273 | 0.348 | 0.149 | 0.417 | 0.153 | 0.166 | 0.131 | 0.167 | 0.069 |
| 646 | 0.387 | 0.473 | 0.464 | 0.609 | 0.736 | 0.447 | 0.53 | 0.486 | 0.617 | 0.316 | 0.604 | 0.249 | 0.34 | 0.237 | 0.278 | 0.144 |
| 651 | 0.208 | 0.295 | 0.339 | 0.323 | 0.608 | 0.264 | 0.259 | 0.259 | 0.42 | 0.153 | 0.45 | 0.136 | 0.171 | 0.118 | 0.169 | 0.073 |
| 660 | 0.362 | 0.464 | 0.454 | 0.573 | 0.735 | 0.437 | 0.541 | 0.496 | 0.625 | 0.292 | 0.589 | 0.238 | 0.311 | 0.225 | 0.282 | 0.123 |
| 669 | 0.2 | 0.289 | 0.325 | 0.33 | 0.628 | 0.272 | 0.259 | 0.253 | 0.439 | 0.157 | 0.456 | 0.148 | 0.165 | 0.117 | 0.167 | 0.071 |
| 673 | 0.421 | 0.615 | 0.521 | 0.668 | 0.758 | 0.456 | 0.581 | 0.494 | 0.715 | 0.424 | 0.659 | 0.274 | 0.342 | 0.245 | 0.345 | 0.158 |
| 707 | 0.294 | 0.387 | 0.408 | 0.528 | 0.722 | 0.403 | 0.387 | 0.366 | 0.514 | 0.234 | 0.524 | 0.25 | 0.275 | 0.207 | 0.206 | 0.115 |
| 728 | 0.065 | 0.003 | 0.205 | 0.151 | 0.406 | 0.16 | 0.101 | 0.133 | 0.008 | 0.004 | 0.311 | NA | 0.106 | 0.075 | 0.004 | 0.031 |
| 737 | 0.201 | 0.237 | 0.33 | 0.317 | 0.582 | 0.302 | 0.247 | 0.275 | 0.371 | 0.148 | 0.454 | NA | 0.206 | 0.158 | 0.137 | 0.086 |
| 746 | 0.418 | 0.545 | 0.567 | 0.723 | 0.788 | 0.618 | 0.694 | 0.604 | 0.636 | 0.434 | 0.679 | NA | 0.551 | 0.379 | 0.314 | 0.172 |
| 752 | 0.567 | 0.728 | 0.727 | 0.885 | 0.877 | 0.772 | 0.845 | 0.789 | 0.775 | 0.65 | 0.802 | 0.485 | 0.706 | 0.533 | 0.452 | 0.256 |
| 768 | 0.584 | 0.787 | 0.748 | 0.895 | 0.877 | 0.804 | 0.86 | 0.82 | 0.803 | 0.712 | 0.803 | 0.523 | 0.78 | 0.574 | 0.499 | 0.288 |
| 782 | 0.166 | 0.197 | 0.313 | 0.281 | 0.483 | 0.283 | 0.376 | 0.245 | 0.322 | 0.152 | 0.419 | 0.151 | 0.228 | 0.165 | 0.15 | 0.073 |
| 811 | 0.371 | 0.436 | 0.474 | 0.532 | 0.746 | 0.539 | 0.569 | 0.531 | 0.635 | 0.423 | 0.607 | 0.317 | 0.431 | 0.28 | 0.327 | 0.16 |
| 836 | 0.747 | 0.782 | 0.872 | 0.942 | 0.943 | 0.912 | 0.943 | 0.938 | 0.905 | 0.837 | 0.895 | 0.732 | 0.9 | 0.8 | 0.722 | 0.487 |
| 844 | 0.504 | 0.494 | 0.584 | 0.736 | 0.807 | 0.707 | 0.806 | 0.734 | 0.722 | 0.453 | 0.69 | 0.427 | 0.629 | 0.465 | 0.422 | 0.222 |
