# Supplementary material for: The expression level of chicken telomerase reverse transcriptase in tumors induced by ALV-J is positively correlated with methylation and mutation of its promoter region
Source: Vet Res. 2022 Jun 23;53:49. doi: 10.1186/s13567-022-01069-2 (PMC9229480; doi:10.1186/s13567-022-01069-2)
Supplement: Supplementary file 4 — Additional file 4. Agarose gel electrophoresis of PCR amplification of the chTERT promoter region. Lanes 1-8: different tissue samples; Lane 9: negative control. [file 13567_2022_1069_MOESM4_ESM.doc]

**Additional file 4**


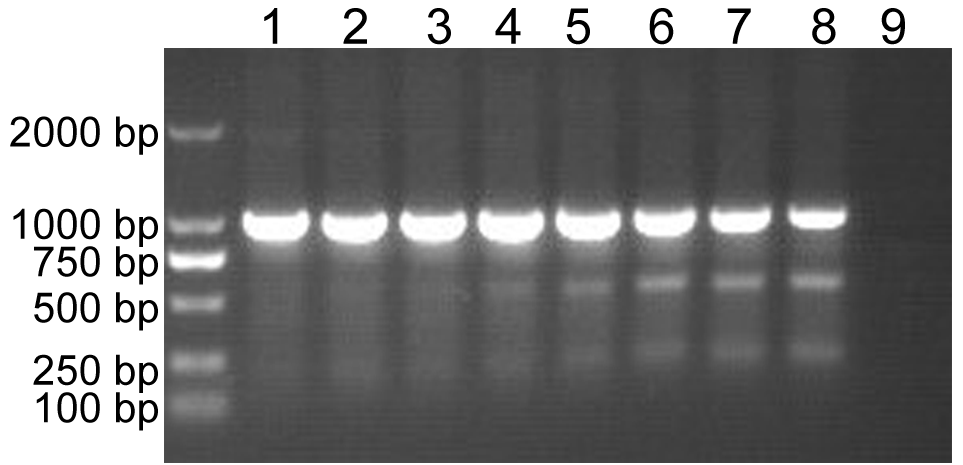


**Agarose gel electrophoresis of PCR amplification of chTERT promoter region.** Lanes 1 8: different tissue samples; Lane 9: negative control.
