# Supplementary material for: The expression level of chicken telomerase reverse transcriptase in tumors induced by ALV-J is positively correlated with methylation and mutation of its promoter region
Source: Vet Res. 2022 Jun 23;53:49. doi: 10.1186/s13567-022-01069-2 (PMC9229480; doi:10.1186/s13567-022-01069-2)
Supplement: Supplementary file 5 — Additional file 5. Analysis of DNA sequence mutations in the promoter region of chTERT. Green box: normal tissues; blue box: tumor-adjacent tissues; red box: tumor tissues; orange box: mutant tumors. T: tumor tissues; TA: tumor-adjacent tissues; N: normal tissues. [file 13567_2022_1069_MOESM5_ESM.doc]

**Additional file 5**


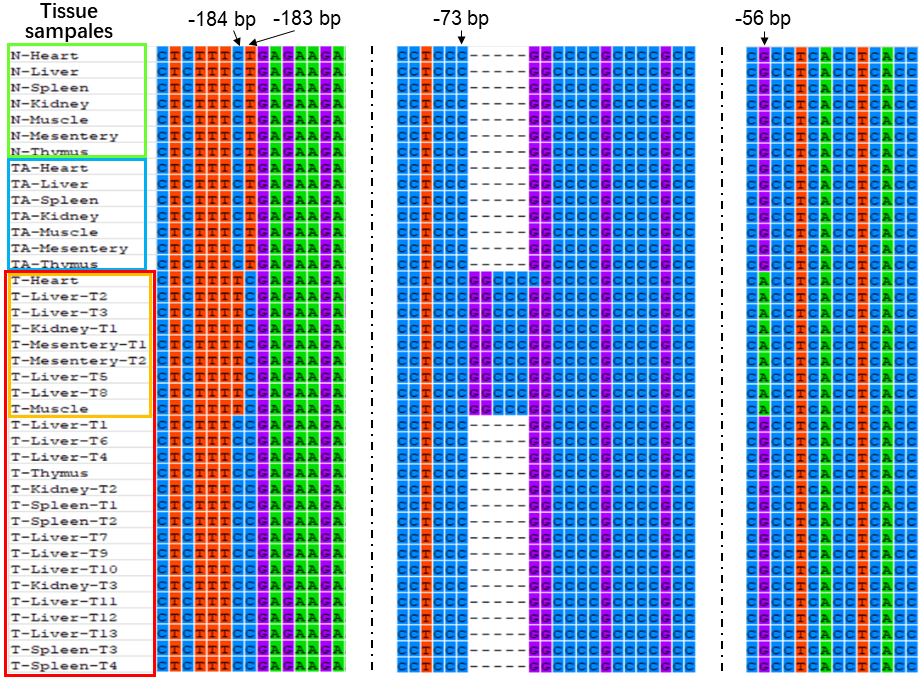


**Analysis of DNA sequence mutations in the promoter region of chTERT.** Green box: normal tissues; blue box: tumor-adjacent tissues; red box: tumor tissues; orange box: mutant tumors. T: tumor tissues; TA: tumor-adjacent tissues; N: normal tissues.
